# Advances in Semen Extenders for Small Ruminant Sperm Preservation: Biological Mechanisms and Emerging Strategies

**DOI:** 10.3390/ani16193104

**Published:** 2026-10-02

**Authors:** Daniel Berean, Liviu Marian Bogdan, Ionela Ut, Raluca Cimpean

**Affiliations:** 1 Department of Reproduction, Faculty of Veterinary Medicine, University of Agricultural Sciences and Veterinary Medicine Cluj-Napoca, Calea Manastur 3-5, 400372 Cluj-Napoca, Romania; liviu.bogdan@usamvcluj.ro (L.M.B.); ionela-delia.ut@student.usamvcluj.ro (I.U.); 2 Academy of Romanian Scientists, Ilfov 3, 050044 Bucharest, Romania; calina-raluca.cimpean@usamvcluj.ro; 3 Department of Animal Breeding and Food Safety, Faculty of Veterinary Medicine, University of Agricultural Sciences and Veterinary Medicine Cluj-Napoca, Calea Manastur 3-5, 400372 Cluj-Napoca, Romania

**Keywords:** semen extenders, semen cryopreservation, ram semen, buck semen, cryoinjury, animal-component-free extenders

## Abstract

Semen preservation represents a fundamental component of reproductive biotechnology in small ruminants, supporting artificial insemination programs, genetic improvement, germplasm conservation, and sustainable livestock production. However, ram and buck spermatozoa are highly susceptible to cryoinjury induced by cold shock, oxidative stress, osmotic imbalance, and membrane destabilization during cooling, freezing, and thawing procedures. Consequently, semen extenders play a critical role in maintaining sperm viability, motility, membrane integrity, mitochondrial activity, and fertilizing capacity during preservation. This narrative review summarizes the biological basis of sperm preservation in sheep and goats and discusses the principal conventional and emerging extender systems used in small ruminant semen cryopreservation. Conventional extender components, including buffering systems, energy substrates, cryoprotective agents, egg yolk, and milk proteins, are reviewed with emphasis on their mechanisms of action and protective effects against cryodamage. Particular attention is given to species-specific limitations associated with buck seminal plasma enzymes and their interactions with egg yolk- and milk-based extenders. In addition, recent advances involving chemically defined extenders, soy lecithin formulations, antioxidants, synthetic membrane stabilizers, and nanotechnology-derived additives are discussed as promising alternatives aimed at improving cryosurvival, reducing oxidative stress, and enhancing extender standardization and biosafety. Practical applications of semen extenders in artificial insemination and semen banking are also discussed together with current methodological limitations, species-specific challenges, and future research priorities. Although numerous emerging strategies have demonstrated promising improvements in laboratory sperm-quality parameters, evidence supporting consistent fertility benefits under field conditions remains limited. Overall, this review critically evaluates current knowledge and highlights the need for species-specific, protocol-oriented, and well-standardized approaches to advance semen preservation in small ruminants.

## Simple Summary

Semen preservation is widely used in sheep and goat reproduction to support artificial insemination, genetic improvement, and conservation of valuable breeds. During cooling and freezing, sperm cells are exposed to several damaging factors such as cold shock, oxidative stress, osmotic imbalance, and ice crystal formation, which reduce sperm quality and fertility. Semen extenders are protective media added to semen to maintain sperm viability during storage and cryopreservation. Conventional extenders commonly contain buffers, sugars, cryoprotectants, egg yolk, or milk proteins that help stabilize sperm membranes and reduce cryodamage. However, biological variability, contamination risks, and negative interactions between goat seminal plasma enzymes and animal-derived extender components remain important limitations. For this reason, recent research has focused on the development of alternative extender systems such as soy lecithin-based formulations, chemically defined media, antioxidants, synthetic membrane stabilizers, and nanotechnology-derived additives. These new approaches aim to improve sperm survival, extender standardization, and biosafety, although many remain under experimental evaluation. This review explains the biological mechanisms underlying semen preservation and discusses the advantages, limitations, and future prospects of conventional and emerging semen extenders for sheep and goats.

## 1. Introduction

Small ruminants, particularly sheep and goats, play a crucial role in global livestock production by contributing significantly to meat, milk, fiber, and skin production, especially in developing and semi-arid regions [1,2]. These species are highly adaptable to harsh environmental conditions and constitute an important source of income and food security for rural communities. In recent decades, the growing demand for animal protein and the need for rapid genetic improvement have increased the importance of assisted reproductive technologies (ARTs), particularly artificial insemination (AI), in small ruminant production systems [3,4,5].

Artificial insemination offers several advantages, including accelerated dissemination of superior genetics, improved breeding management, disease control, and reduced costs associated with maintaining breeding males. However, the success of AI programs largely depends on the ability to preserve semen quality during short- and long-term storage [6,7]. Semen preservation technologies, including liquid storage and cryopreservation, are therefore essential tools for reproductive management and germplasm conservation in sheep and goats.

Depending on the intended duration of storage, semen preservation can be broadly classified into chilled (liquid) preservation and cryopreservation. Chilled semen is generally stored at 4–15 °C for short-term use, allowing maintenance of sperm viability for several days while avoiding freezing-associated damage. In contrast, cryopreservation involves storage in liquid nitrogen (−196 °C) for long-term genetic conservation but exposes spermatozoa to additional stressors such as intracellular ice crystal formation, cryoprotectant toxicity, and freeze–thaw injury. Although both preservation methods share several biological challenges, including cold shock and oxidative stress, the extent and mechanisms of sperm damage differ considerably. Throughout this review, these preservation systems are discussed separately whenever their biological responses or extender requirements differ [5,7]. Despite substantial progress in semen preservation techniques, the cryobiology of small ruminant spermatozoa remains highly challenging. Ram and buck sperm cells are particularly susceptible to various forms of cellular damage during cooling, freezing, and thawing processes. These procedures can induce cold shock, osmotic stress, oxidative damage, membrane destabilization, mitochondrial dysfunction, DNA fragmentation, and acrosomal injury, ultimately reducing sperm viability and fertilizing capacity [8,9]. Furthermore, species-specific differences complicate semen preservation protocols, particularly in goats, where seminal-plasma interactions with extender components can influence sperm survival during processing and preservation. Recent multi-omics analyses of buck semen have also demonstrated that cryopreservation is associated with coordinated molecular and metabolic alterations [10].

To minimize cryoinjuries and maintain sperm functionality, semen extenders are routinely used during semen processing and preservation. Semen extenders are specialized media formulated to provide nutrients, maintain osmotic and pH balance, protect sperm membranes against thermal and oxidative stress, and enhance sperm survival during storage [11]. Conventional extenders commonly contain buffering agents, energy substrates, cryoprotectants, antibiotics, and membrane stabilizers such as egg yolk or milk derivatives. Over time, advances in reproductive biotechnology have led to the development of more sophisticated extender formulations incorporating antioxidants, amino acids, plant extracts, nanoparticles, and chemically defined compounds aimed at improving post-thaw sperm quality and fertility outcomes [11,12,13].

In recent years, increasing attention has been directed toward the replacement of animal-derived extender components due to concerns regarding biosecurity, biological variability, and microbial contamination. Consequently, formulations based on soy lecithin, liposomes, and synthetic cryoprotective agents have been investigated as alternatives for small ruminant semen preservation [14,15]. Natural antioxidants and nanotechnology-based additives have also been evaluated as approaches to reduce oxidative stress and improve cryotolerance, although the level of evidence varies considerably among compounds and species [16].

Although numerous studies have investigated the effects of different extender compositions on semen quality in sheep and goats, direct comparison among studies remains difficult because of differences in species, breed, season, extender composition, additive concentration, seminal plasma management, cryopreservation protocols, and sperm evaluation methods. Consequently, some apparently conflicting results may reflect methodological variation rather than true biological differences.

Accordingly, this narrative review aims to critically discuss the composition, biological functions, and recent advances in semen extenders used for the preservation of small ruminant semen, with particular emphasis on ram and buck sperm cryobiology, antioxidant supplementation, and emerging technologies for improving sperm survival and fertility potential.

## 2. Methods

The manuscript was prepared as a narrative review to summarize and critically evaluate the current knowledge on semen extenders and preservation strategies in small ruminants, with emphasis on sheep and goats.

A comprehensive literature search was performed using the electronic databases PubMed, Scopus, Web of Science, and Google Scholar. The primary literature search was conducted in May 2025 and included publications from 1990 to May 2025. Subsequently, during manuscript preparation and revision, the literature was monitored for newly published relevant studies to maintain the currency of the review.

The following search terms and Boolean operators were used in different combinations: (“ram semen” OR “buck semen” OR sheep OR goat) and (“semen extender” OR “semen preservation” OR cryopreservation OR chilled semen OR liquid storage OR “artificial insemination”) and (egg yolk OR milk OR soy lecithin OR antioxidant OR liposome OR nanoparticle OR “chemically defined extender” OR cryoprotectant OR proteomics OR metabolomics OR lipidomics OR “artificial intelligence” OR exosome OR prostasome).

The retrieved literature was screened for relevance to the scope of this narrative review. Title and abstract screening was followed by full-text assessment when necessary. Priority was given to peer-reviewed original studies involving ram and buck semen preservation; studies in other species were retained only when they provided clearly identified mechanistic or translational information. No formal risk-of-bias or quality-scoring instrument was applied, consistent with the narrative-review design. During the revision process, the structured database search was formally updated through July 2026 using the same databases and search terms. Following completion of this formal search update, targeted literature surveillance continued during manuscript revision and peer review. A limited number of highly relevant publications appearing after July 2026 were subsequently incorporated to maintain the currency of the narrative review. These post-cutoff publications were therefore identified through targeted literature surveillance rather than through the formal July 2026 database search.

Only publications written in English were considered. Priority was given to peer-reviewed original research articles involving ram and buck semen preservation. Previous review articles were consulted primarily to provide historical background and identify additional primary references but were not used as the principal source of evidence when original studies were available.

Studies were included if they evaluated semen extenders, extender components, cryoprotective agents, antioxidants, membrane stabilizers, nanotechnology-based additives, or other preservation strategies relevant to chilled or cryopreserved semen in sheep and goats. Studies performed in other livestock species were included only when they provided mechanistic information or emerging technologies with clear translational relevance to small ruminants. Publications lacking sufficient methodological detail, conference abstracts, non-peer-reviewed literature, or studies unrelated to semen preservation were excluded.

For the purposes of this review, conventional technologies were defined as extender systems routinely used in reproductive practice, including Tris-, citrate-, or milk-based formulations supplemented with egg yolk, glycerol, and other established cryoprotective components. Emerging technologies included chemically defined extenders, plant-derived formulations, synthetic membrane stabilizers, nanotechnology-based additives, omics-guided approaches, artificial intelligence-assisted semen evaluation, and extracellular vesicle-based preservation strategies. The selected literature was synthesized narratively, with particular emphasis on comparing species-specific responses, identifying knowledge gaps, critically evaluating conflicting findings, and highlighting future research directions.

## 3. Biology of Small Ruminant Sperm Preservation

Successful semen preservation in small ruminants depends largely on understanding the biological and physiological responses of spermatozoa to cooling, liquid storage, freezing, and thawing procedures. Sperm cells are highly specialized and structurally complex cells with limited cytoplasmic content and reduced antioxidant defense mechanisms, making them particularly vulnerable to environmental and physicochemical stressors during preservation. In sheep and goats, spermatozoa exhibit considerable sensitivity to cold shock, osmotic imbalance, oxidative stress, and cryoinjury, all of which negatively affect post-preservation sperm quality and fertility [9,17,18].

Mammalian spermatozoa possess a highly specialized structure in which the plasma membrane is particularly rich in polyunsaturated fatty acids (PUFAs), maintaining membrane fluidity but also increasing susceptibility to lipid peroxidation and cryoinjury during semen preservation. Furthermore, the mitochondrial sheath located in the sperm midpiece provides the ATP required for motility, making mitochondrial integrity essential for post-preservation sperm function [19,20,21].

Unlike somatic cells, spermatozoa possess minimal cytoplasmic volume and limited endogenous antioxidant systems [11]. Consequently, sperm cells have reduced capacity to repair structural and molecular damage induced during cryopreservation. Damage to the plasma membrane, acrosome, mitochondria, or DNA may compromise sperm motility, viability, and fertilizing ability [22,23].

Cold shock is one of the primary causes of sperm damage during semen preservation, particularly in ram and buck semen [24]. It occurs when spermatozoa are exposed to rapid temperature reduction, leading to abrupt alterations in membrane lipid organization and permeability. During cooling, membrane phospholipids undergo phase transitions from a fluid to a gel state, resulting in loss of membrane integrity and altered membrane protein function. These structural modifications may cause leakage of intracellular components, disruption of ion transport systems, reduced enzyme activity, and impaired mitochondrial function. In severe cases, cold shock can lead to irreversible membrane rupture and sperm cell death. In ram spermatozoa, cold-shock-induced membrane alterations and the protective influence of seminal-plasma proteins against such damage have been experimentally demonstrated [25,26].

During chilled semen preservation, spermatozoa remain metabolically active despite reduced temperatures. Cooling decreases metabolic activity and ATP consumption, thereby prolonging sperm survival; however, prolonged liquid storage may still induce membrane destabilization, oxidative stress, and bacterial proliferation. Therefore, extenders intended for chilled storage are primarily designed to stabilize plasma membranes, maintain osmotic balance, provide metabolic substrates, and limit oxidative damage, preserving sperm functionality for short-term artificial insemination [5,22].

Cryopreservation exposes spermatozoa to substantially greater physicochemical stress than chilled storage. In addition to cold shock and oxidative stress, freezing and thawing induce extracellular and intracellular ice crystal formation, osmotic dehydration, cryoprotectant toxicity, membrane phase transitions, mitochondrial dysfunction, and increased reactive oxygen species production, which may ultimately result in DNA fragmentation and irreversible acrosomal damage. Consequently, cryopreservation requires specialized extenders containing penetrating and non-penetrating cryoprotectants, together with membrane stabilizers such as egg yolk phospholipids, low-density lipoproteins (LDL), or milk proteins, to minimize ice formation, reduce osmotic injury, preserve membrane integrity, and maintain fertilizing capacity during freezing and thawing [25,27].

Oxidative stress is another major factor limiting sperm survival during preservation. Reactive oxygen species (ROS), including superoxide anions, hydrogen peroxide, and hydroxyl radicals, are naturally generated during sperm metabolism. Under physiological conditions, low levels of ROS are necessary for sperm capacitation, hyperactivation, and fertilization. However, excessive ROS production during semen handling and cryopreservation overwhelms antioxidant defense systems and induces oxidative damage [11].

The high concentration of polyunsaturated fatty acids in sperm membranes makes small ruminant spermatozoa highly vulnerable to lipid peroxidation. Oxidative degradation of membrane lipids results in reduced membrane fluidity, increased permeability, mitochondrial dysfunction, DNA fragmentation, and decreased sperm motility. Additionally, oxidative stress may impair acrosomal integrity and fertilization capacity [28,29,30].

Cryopreservation procedures further exacerbate ROS generation because of temperature fluctuations, mitochondrial dysfunction, and reduced antioxidant protection. Excessive oxidative stress contributes to lipid peroxidation, membrane destabilization, impaired mitochondrial function, DNA damage, and reduced sperm viability. Consequently, supplementation of extenders with enzymatic and non-enzymatic antioxidants has been extensively investigated as a strategy to reduce oxidative injury during sperm preservation [11,28,29,30].

Spermatozoa are exposed to significant osmotic stress during addition and removal of cryoprotective agents as well as during freezing and thawing. Changes in extracellular osmolarity induce rapid movement of water across the sperm membrane, leading to cell shrinkage or swelling. Excessive osmotic fluctuations may disrupt membrane integrity, alter intracellular ion concentrations, and impair cellular metabolism [18,31].

During freezing, extracellular ice formation increases solute concentration in the unfrozen fraction, causing dehydration of sperm cells. Although controlled dehydration is necessary to minimize intracellular ice crystal formation, excessive water loss can damage membrane proteins and cytoskeletal structures. Conversely, rapid rehydration during thawing may lead to membrane rupture and loss of sperm viability. The extent of osmotic injury depends on cooling rate, cryoprotectant concentration, and species-specific membrane permeability characteristics. Therefore, extender formulations must carefully balance osmolarity and cryoprotective capacity to maintain sperm survival [32,33,34].

Cryoinjury refers to the cumulative structural and functional damage sustained by spermatozoa during freezing and thawing. This damage arises from a combination of cold shock, osmotic stress, oxidative injury, and intracellular ice crystal formation. Cryopreservation can adversely affect multiple sperm structures, including the plasma membrane, acrosome, mitochondria, cytoskeleton, and nuclear DNA [35]. Mitochondrial dysfunction is particularly important because reduced ATP production directly impairs sperm motility. Furthermore, DNA fragmentation induced by oxidative stress and apoptosis-like processes may compromise embryo development and fertility even when sperm motility appears normal [36,37].

Acrosomal damage is another critical consequence of cryoinjury. The acrosome contains hydrolytic enzymes required for penetration of the zona pellucida during fertilization. Structural disruption of the acrosomal membrane reduces fertilizing capacity and decreases pregnancy rates following artificial insemination [38].

Although ram and buck spermatozoa share many physiological characteristics, important species-specific differences influence semen preservation outcomes. In goats, bulbourethral secretions contain enzymatic activities that can interact adversely with animal-derived extender components. Historically, the activity responsible for deterioration in egg-yolk-containing media has been described as the egg-yolk coagulating enzyme (EYCE); classical biochemical work associated this effect with phospholipase A activity and hydrolysis of egg-yolk phospholipids, with formation of lysophospholipids and free fatty acids [39,40]. Milk-related toxicity has been associated with a bulbourethral 60 kDa glycoprotein exhibiting triglyceride-lipase activity [40]. In milk-based extenders, hydrolysis of residual milk triglycerides releases free fatty acids, particularly oleic acid, which can impair sperm motility and viability [40]. Because terminology and enzyme identity have not always been used consistently in the literature, the effects observed in egg-yolk- and milk-based extenders are best described according to the substrate hydrolyzed and the experimentally demonstrated biological outcome rather than by treating historical enzyme terminology as interchangeable. These interactions help explain why dilution or removal of seminal plasma is used in some buck semen-processing protocols, although the processing procedure itself must be optimized to avoid additional sperm damage [40,41].

**Figure 1:**
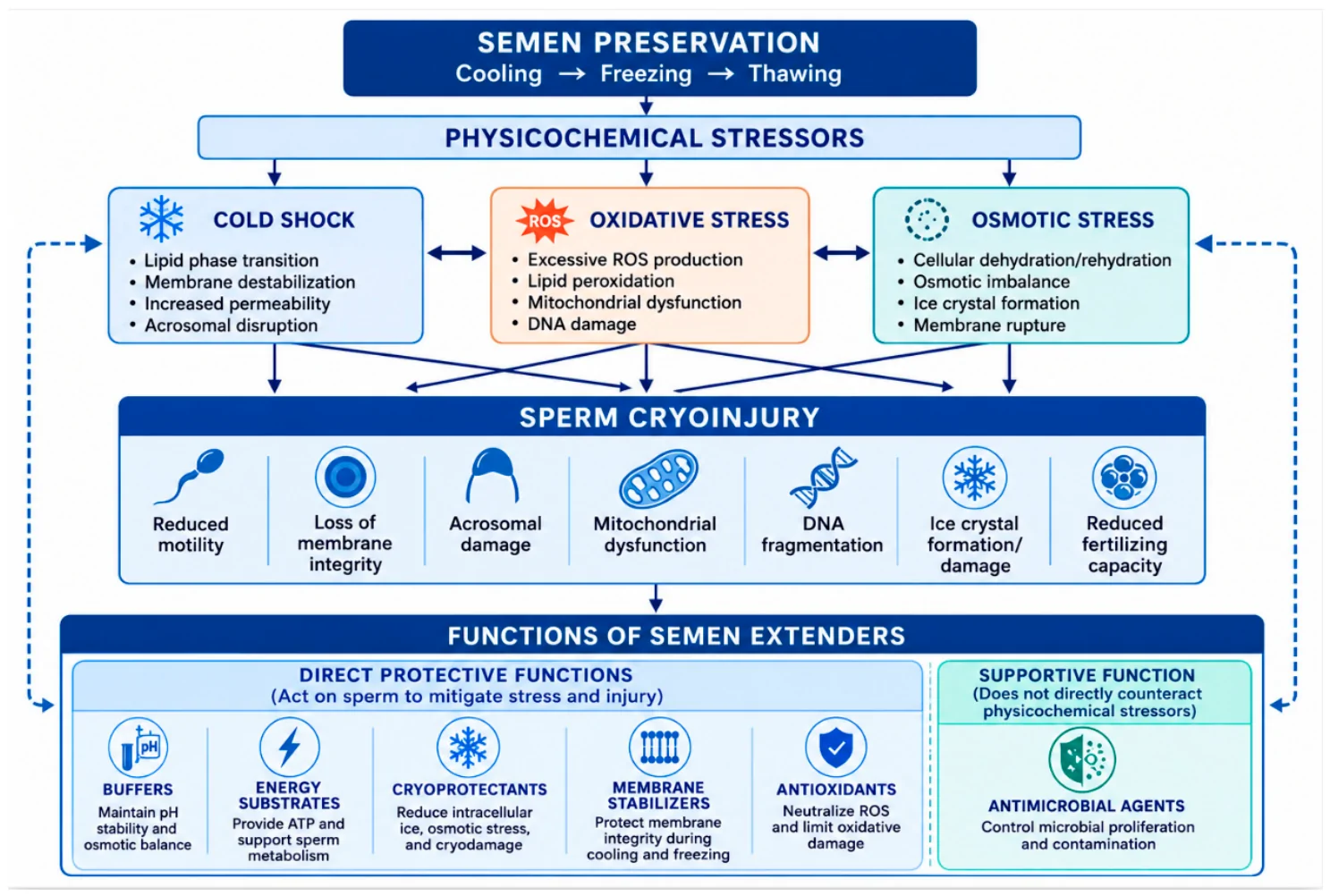
Workflow illustrating the principal biological stress responses occurring during semen preservation in small ruminants and the protective mechanisms provided by semen extenders. Cooling, freezing, and thawing procedures induce cold shock, oxidative stress, and osmotic imbalance, leading to membrane destabilization, mitochondrial dysfunction, DNA fragmentation, and reduced fertilizing capacity. Extender components such as buffers, cryoprotectants, antioxidants, and membrane stabilizers contribute to the maintenance of sperm viability and functional integrity during preservation.

Collectively, understanding the biological mechanisms underlying sperm injury during preservation is fundamental for the development of efficient semen extenders capable of protecting spermatozoa against thermal, osmotic, and oxidative stress. Such knowledge provides the foundation for the design of advanced preservation strategies aimed at improving fertility outcomes in small ruminants (Figure 1).

## 4. Semen Extenders: Definition, Biological Roles, and Functional Importance in Small Ruminant Semen Preservation

Semen preservation is a fundamental component of assisted reproductive technologies in small ruminants, and the success of preservation procedures largely depends on the composition and efficacy of semen extenders. During both chilled storage and cryopreservation, spermatozoa are exposed to different physicochemical stressors that compromise cellular integrity. However, whereas chilled preservation mainly aims to maintain sperm metabolism and membrane stability during short-term storage, cryopreservation additionally requires protection against freeze–thaw injury, intracellular ice formation, and cryoprotectant-associated toxicity. Consequently, extender composition should be adapted according to the intended preservation strategy [42,43].

Semen extenders are complex biological media designed to preserve sperm viability, motility, membrane integrity, and fertilizing potential during in vitro storage by supplying nutrients, maintaining osmotic and pH stability, protecting against oxidative and thermal stress, and preventing microbial contamination. These extenders dilute ejaculated semen while simultaneously minimizing cellular damage induced by cooling, freezing, and thawing processes [44].

An ideal semen extender should possess several essential characteristics, including adequate buffering capacity, isotonicity, low toxicity, cryoprotective properties, antimicrobial activity, and compatibility with sperm metabolism. Moreover, extender formulations should maintain the structural integrity of the plasma membrane and acrosome while preserving mitochondrial activity and DNA stability [45,46,47].

### 4.1. Main Functions of Semen Extenders

Spermatozoa require a continuous energy supply to sustain motility, membrane transport systems, and metabolic activities during preservation. Because sperm cells possess limited intracellular reserves, extenders are supplemented with metabolizable substrates such as fructose, glucose, and lactose that support ATP production through glycolysis and oxidative phosphorylation [48]. Adequate nutrient availability is particularly important during liquid storage, where sperm metabolism remains active for extended periods.

Sperm metabolism generates acidic by-products, including lactic acid, which may reduce pH and impair sperm function during storage. Therefore, semen extenders contain buffering systems that maintain physiological pH and osmotic equilibrium. Stabilization of the extracellular environment is essential for preserving membrane integrity, enzymatic activity, and ion transport processes [49,50].

The sperm plasma membrane is highly susceptible to thermal and osmotic stress due to its elevated polyunsaturated fatty acid content. Membrane stabilizers such as egg yolk phospholipids, low-density lipoproteins (LDL), and milk proteins interact with sperm membranes to preserve membrane fluidity and reduce cold shock-induced damage during cooling and freezing [51].

Cryoprotective agents are among the most important components of semen extenders. These compounds reduce cellular injury associated with ice crystal formation, osmotic imbalance, and dehydration during cryopreservation. Cryoprotectants function by stabilizing intracellular water dynamics, lowering the freezing point, and minimizing mechanical damage during freezing and thawing [52]. In addition, cryopreservation enhances the generation of reactive oxygen species (ROS), leading to oxidative stress and lipid peroxidation. Therefore, extenders intended for cryopreservation are frequently supplemented with antioxidants to neutralize ROS and protect sperm structures, including the plasma membrane, mitochondria, and DNA, from oxidative injury [11].

Semen collection and processing procedures inevitably expose samples to bacterial contamination. Microbial proliferation may reduce sperm viability through toxin production, nutrient depletion, and pH alteration. Therefore, antibiotics are routinely incorporated into extenders to inhibit bacterial growth and maintain semen quality during storage [53]. In commercial semen preservation, antibiotics are generally incorporated as combinations of broad-spectrum agents, such as penicillin–streptomycin, gentamicin, or lincomycin–spectinomycin, to provide activity against both Gram-positive and Gram-negative bacteria. Although these compounds contribute substantially to microbiological safety, their efficacy depends on appropriate concentration, as excessive antibiotic exposure may impair sperm motility, membrane integrity, mitochondrial activity, and overall sperm function [54]. In addition, the routine use of antibiotics in semen extenders has raised increasing concerns regarding the emergence of antimicrobial resistance and has prompted greater regulatory attention to prudent antimicrobial use in animal reproduction. Consequently, recent research has focused on complementary or alternative antimicrobial strategies, including antimicrobial peptides, plant-derived bioactive compounds, nanoparticles, improved hygienic semen collection procedures, and optimized semen-processing protocols designed to minimize bacterial contamination while reducing reliance on conventional antibiotics. Although many of these approaches have shown promising results under experimental conditions, further validation is required before their routine application in commercial semen preservation [53,54,55].

### 4.2. Conventional Semen Extenders

Conventional semen extenders are composed of multiple interacting constituents specifically designed to protect spermatozoa against the detrimental effects associated with cooling, liquid storage, and cryopreservation. The overall efficacy of an extender depends not only on the individual properties of its components but also on their synergistic interactions. However, the relative contribution of each component is difficult to establish because most studies evaluate complete extender formulations rather than individual ingredients under standardized experimental conditions. Among the most important constituents of semen extenders are buffering agents, energy substrates, and cryoprotective compounds. Buffers play a fundamental role in maintaining pH stability and osmotic balance during semen preservation, as spermatozoa are highly sensitive to fluctuations in hydrogen ion concentration and osmolarity. Even minor deviations from physiological pH may impair mitochondrial activity, membrane permeability, enzymatic reactions, and sperm motility. Commonly used buffering systems in small ruminant semen extenders include Tris (tris(hydroxymethyl)aminomethane), citrate, and phosphate-based formulations.

Tris-based extenders are widely used in ram and buck semen preservation because of their effective buffering capacity within the pH range relevant to sperm preservation [56,57]. However, the pH of Tris-containing solutions is temperature-dependent; therefore, buffering capacity should not be interpreted as temperature-independent pH stability. The measured pH should be considered in relation to the temperature and the complete extender formulation. Within complete extender formulations, Tris contributes to control of the extracellular environment during preservation, while resulting sperm-quality outcomes depend on the combined effects of the buffer, cryoprotectants, membrane-protective components, and preservation protocol [56,57,58,59]. Consequently, Tris–egg yolk–glycerol formulations remain among the most extensively employed extenders in ram semen cryopreservation [58,59].

Citrate-based buffers also contribute significantly to sperm preservation by acting both as buffering agents and chelators of divalent ions such as calcium, thereby regulating ionic balance, stabilizing sperm membranes, and reducing premature capacitation during storage. In addition, citrate contributes to osmolarity maintenance and supports sperm metabolism, although its buffering efficiency under prolonged storage conditions is generally lower than that of Tris-based systems [56]. Phosphate-buffered extenders are commonly applied for short-term semen preservation due to their physiological compatibility and satisfactory pH stabilization; however, phosphate systems may interact negatively with certain extender constituents during freezing, limiting their effectiveness in long-term cryopreservation protocols. Beyond pH regulation, buffering agents play a critical role in maintaining isotonic conditions within the preservation medium, thereby preventing excessive sperm swelling or shrinkage that may compromise membrane integrity, ion transport systems, and mitochondrial function [57].

In parallel with buffering systems, semen extenders contain energy substrates that sustain sperm metabolism and ATP production throughout storage. Because spermatozoa possess limited endogenous energy reserves, exogenous carbohydrates are required to maintain motility, membrane transport activity, and fertilizing ability [60]. ATP generation in preserved spermatozoa is supported by both glycolysis, occurring predominantly in the principal piece, and oxidative phosphorylation within the mitochondria of the sperm midpiece. However, the relative contribution of these metabolic pathways is not fixed and may vary according to species, substrate availability, extender composition, incubation conditions, and preservation protocol. Adequate energy production is essential for maintaining sperm motility, ion homeostasis, membrane integrity, and fertilization capability; nevertheless, excessive metabolic activity may accelerate ROS generation and reduce sperm longevity, emphasizing the need for balanced extender formulations capable of sustaining metabolism while minimizing oxidative stress [61,62]. Among the carbohydrates incorporated into semen extenders, fructose is one of the principal physiological energy substrates available to small-ruminant spermatozoa and serves as a readily metabolizable substrate for glycolysis. However, substrate utilization and the relative dependence on glycolytic versus oxidative metabolism may differ between ram and buck spermatozoa and are influenced by species, extender formulation, substrate availability, storage conditions, and incubation time. Supplementation with fructose has been associated with improved progressive motility and enhanced post-thaw survival in ram semen; however, the magnitude of this benefit varies according to species, preservation protocol, and the presence of other extender components. Glucose also serves as an important energy source and contributes to osmotic balance within extender formulations; however, excessive glucose concentrations may stimulate elevated metabolic activity and reactive oxygen species (ROS) production during prolonged storage, highlighting the importance of optimizing carbohydrate concentration rather than assuming that higher concentrations are universally beneficial. Lactose is frequently incorporated into cryopreservation extenders because of its combined function as an energy substrate and osmotic regulator. In addition to supporting cellular metabolism, lactose contributes to membrane stabilization during freezing and reduces intracellular ice formation through osmotic modulation [60,61,62].

Cryoprotective agents represent another indispensable category of extender components used specifically during semen cryopreservation. Their primary function is to protect spermatozoa against freezing-induced damage by reducing intracellular ice crystal formation, osmotic stress, membrane destabilization, and excessive cellular dehydration. According to their mechanism of action, cryoprotectants are generally classified into penetrating and non-penetrating compounds based on their ability to permeate the sperm plasma membrane [63].

Among penetrating cryoprotectants, glycerol remains the most extensively utilized in small ruminant semen cryopreservation due to its low molecular weight and high membrane permeability, allowing it to replace intracellular water molecules and thereby minimize intracellular ice formation during freezing. Glycerol also lowers the freezing point of the medium and reduces solution effects associated with cryopreservation; however, excessive glycerol concentrations may exert cytotoxic effects and alter membrane permeability, particularly in goat spermatozoa [63,64]. Dimethyl sulfoxide (DMSO) possesses high membrane permeability and effective cryoprotective properties against intracellular ice formation and osmotic injury, although its use in small ruminants is limited by potential toxicity at elevated concentrations [65]. Ethylene glycol, characterized by rapid cellular penetration and lower viscosity compared with glycerol, has also demonstrated promising effects on post-thaw sperm quality, although species-specific responses remain variable [66]. In contrast, non-penetrating cryoprotectants such as trehalose and sucrose exert their protective effects extracellularly by promoting controlled cellular dehydration and reducing osmotic stress during freezing and thawing. Trehalose interacts with membrane phospholipids, preserving membrane fluidity and stability, whereas sucrose contributes to osmotic regulation and reduction in intracellular ice formation, thereby improving membrane integrity and acrosomal preservation [67,68]. Ultrastructural alterations associated with ram sperm cryopreservation have also been documented and provide additional morphological evidence of freeze–thaw-related sperm damage [69] (Table 1).

## 5. Egg Yolk-Based Extenders

Egg yolk-based extenders have been applied to both chilled semen preservation and cryopreservation in small ruminants. During cooling, egg-yolk constituents contribute to membrane stabilization and protection against cold shock, whereas during cryopreservation they also help limit membrane damage associated with freezing and thawing [24,25,71,72,73,74]. The protective effect is mainly attributed to phospholipids, cholesterol, and low-density lipoprotein (LDL) fractions that interact with the sperm surface and influence membrane stability during temperature change [72,73,74]. In ram semen, egg-yolk-containing extenders have been associated with preservation of post-thaw motility, membrane and acrosomal integrity and, in selected studies, fertility after insemination; however, the magnitude of these effects depends on extender composition and the freezing and insemination protocols [5,71,72]. Thus, Tris-egg yolk-glycerol formulations remain useful conventional comparators, but laboratory improvements should not automatically be interpreted as evidence of superior field fertility.

Purified LDL fractions have been investigated as an alternative to whole egg yolk in order to reduce undefined particulate material while retaining lipid-mediated membrane protection. Studies in ram semen and buck semen indicate that LDL-containing formulations can preserve post-thaw sperm quality under specific protocols [72,74]. Purification may also improve extender transparency and facilitate laboratory assessment compared with whole egg yolk. Nevertheless, the optimal LDL concentration and the extent to which laboratory benefits translate into fertility outcomes remain protocol- and species-dependent.

Commercial formulations should be classified according to the lipid source reported for the specific product rather than grouped solely by brand name. In published ram studies, Biladyl^®^ A has been described as an egg-yolk-based extender, whereas OviXcell^®^ has been described as a soybean-lecithin-based extender. OviXcell^®^ is therefore discussed in the plant-based section of this review and is not classified as an egg-yolk extender. Because commercial formulations may be proprietary or modified over time, product classification in this review is limited to compositions explicitly described in the cited literature [75,76,77,78].

Despite their widespread application and protective effectiveness, egg yolk-based extenders possess several important limitations that have stimulated continuous research into alternative preservation systems. One of the major drawbacks is the considerable variability in egg yolk composition among batches, which may influence extender consistency and preservation outcomes. Variations in phospholipid and cholesterol concentrations related to hen diet, age, breed, and storage conditions can affect sperm cryotolerance and reduce reproducibility between laboratories. In addition, egg yolk presents potential hygienic and biosecurity concerns due to the possibility of microbial contamination and transmission of pathogenic microorganisms [73,79]. The presence of particulate matter within egg yolk may also interfere with CASA analyses and microscopic assessment by reducing sample transparency and creating imaging artifacts. Furthermore, animal-derived biological products face increasing regulatory scrutiny due to biosafety considerations and the demand for chemically defined preservation media [80].

In goat semen preservation, egg-yolk-containing extenders present a species-specific challenge because seminal-plasma enzymatic activity can hydrolyze egg-yolk phospholipids. Biochemical characterization of the major goat seminal-plasma bulbourethral lipase identified it as pancreatic lipase-related protein 2 (GoPLRP2) and demonstrated both triglyceride-lipase and phospholipase A activities [81]. Historical studies of the egg-yolk coagulating enzyme (EYCE) described hydrolysis of egg-yolk constituents with formation of lysophospholipids and free fatty acids, which can adversely affect sperm viability [82,83]. Thus, although historical terminology differs among studies, the biochemical activities should be interpreted according to the substrate involved. For clarity, the present review distinguishes the egg-yolk-associated effect by hydrolysis of phospholipid substrates from the milk-associated effect arising primarily from hydrolysis of residual milk triglycerides. Dilution or removal of seminal plasma before freezing can reduce these interactions, but processing conditions should be optimized because excessive centrifugation may itself damage spermatozoa [40,82,83].

Several strategies have been investigated to reduce the limitations of whole egg yolk. These include partial or complete replacement with purified LDL fractions, antioxidant supplementation, cholesterol-loaded cyclodextrins, and lipid-based alternatives. In ram semen, vitamin E/Trolox supplementation has been evaluated in Tris-egg yolk systems, with effects on post-thaw structure and kinematics that depend on concentration and protocol [84,85]. Cholesterol-loaded cyclodextrins can increase membrane cholesterol and improve resistance to cold shock under defined conditions [86]. Liposome-based formulations have also been evaluated directly in ram semen as alternatives to egg yolk, but their performance remains dependent on formulation, preservation method, and experimental endpoint [73].

Despite their proven efficacy, egg yolk-based extenders present limitations related to biological variability, potential microbial contamination, and species-specific interactions, particularly in buck semen due to phospholipase activity. These limitations have stimulated the development of alternative preservation systems, including purified LDL fractions, soy lecithin-based extenders, chemically defined media, liposomes, nanoparticles, and other synthetic membrane stabilizers [87,88,89].

## 6. Milk-Based Extenders

Milk-based extenders can be used for chilled semen preservation and, in some protocols, cryopreservation. Their protective effects are mainly associated with milk proteins, including casein-containing fractions, together with buffering and osmotic components. In ram semen, milk-based extenders have been evaluated directly during liquid storage [90]. Evidence from deer semen is retained only as cross-species context regarding extender-dependent sperm preservation responses [91]. Direct field evidence in sheep has compared milk- and Tris-based extenders following artificial insemination [92]. Similarly, evidence from buffalo semen is considered only as cross-species context and not as direct ovine evidence [93]. Additional ovine studies have evaluated milk-based formulations during chilled storage and artificial insemination [94,95]. Their application in buck semen requires particular caution because bulbourethral lipase activity can hydrolyze residual milk triglycerides and release toxic free fatty acids [40]. Thus, the suitability of milk-based formulations depends on species, milk processing, seminal-plasma management, and the preservation protocol.

Skim-milk-based extenders have a long history of use because of their accessibility and their ability to support sperm survival during liquid storage. In sheep, controlled studies have compared milk-based and Tris-based systems using laboratory sperm-quality endpoints and field fertility after vaginal or cervical insemination [90,92,94,95]. For example, Paulenz et al. reported higher fertility with cooled liquid ram semen diluted in a milk-based extender than with a Tris-based extender under their field conditions [92], whereas Olivera-Muzante et al. reported comparable conception rates for chilled semen preserved in UHT-milk- and INRA 96^®^-based systems containing egg yolk [94]. These results illustrate that performance depends on formulation, storage duration, and insemination conditions rather than on the generic category “milk-based extender” alone.

Commercial milk-based extenders have also gained widespread use in veterinary reproductive biotechnology. Products such as INRA 96^®^, originally developed by the French National Institute for Agriculture, Food, and Environment (INRAE), have been successfully utilized for liquid semen preservation in several species due to their chemically balanced composition and high preservation efficiency [94]. INRAE formulations contain milk proteins combined with buffering agents, sugars, and antibiotics designed to optimize sperm longevity and membrane stability during storage. Although initially developed for stallion semen, modified milk-protein extenders have also been investigated in small ruminant semen preservation protocols [95]. Similarly, commercially standardized skim milk-based extenders are frequently used in artificial insemination centers because they provide greater reproducibility and reduced preparation variability compared with homemade formulations.

The principal species-specific limitation of milk-based extenders in goats is the interaction between buck seminal-plasma lipase activity and residual milk triglycerides. GoPLRP2, the major bulbourethral lipase characterized in goat seminal plasma, exhibits triglyceride-lipase activity that can hydrolyze milk triglycerides and release free fatty acids; oleic acid has been identified as an important mediator of the associated decline in sperm motility and viability [40,81,96]. This milk-associated mechanism is therefore described according to its triglyceride substrate and should be distinguished from the phospholipid hydrolysis reported in the historical egg-yolk coagulating-enzyme literature.

To minimize the adverse effects of seminal plasma enzymes, centrifugation and removal of seminal plasma prior to extender addition are commonly recommended in goat semen cryopreservation protocols. Washing procedures substantially reduce phospholipase and lipase activity and improve compatibility between spermatozoa and milk-derived extender components. Several studies have demonstrated that removal of seminal plasma prior to cryopreservation significantly enhances post-thaw motility, membrane integrity, and acrosomal preservation in buck semen [97,98,99]. Nevertheless, excessive centrifugation may induce mechanical damage, oxidative stress, and membrane disruption, emphasizing the importance of optimizing centrifugation force and duration. In some protocols, partial seminal plasma removal rather than complete elimination has been proposed to maintain beneficial seminal plasma factors while minimizing enzymatic hydrolysis [100].

Research on milk-based systems has also evaluated antioxidant supplementation and membrane-targeted additives. Plant-derived antimicrobial or antioxidant compounds have been tested directly in buck semen under chilled or frozen-storage conditions [101,102], while broader small-ruminant evidence indicates that antioxidant effects are strongly dose- and formulation-dependent [11]. Cholesterol-loaded cyclodextrins represent a separate membrane-modifying strategy and have been studied directly in ram and goat semen [17]. These interventions should therefore be interpreted according to the additive, species, preservation method, and endpoint rather than as uniformly beneficial modifications of milk-based extenders.

Alternatives to animal-derived milk formulations include chemically defined media and soy-lecithin-based extenders, which have been evaluated in both ram and buck semen under liquid-storage and cryopreservation conditions [23,46,88,89,103,104,105]. Nanoparticle additives have also been evaluated experimentally in small-ruminant semen, but these approaches should be considered separately from conventional membrane stabilizers because their mechanisms and safety profiles differ [106,107]. Compared with egg yolk, milk-based formulations may offer greater optical clarity, whereas their biological performance varies with species, milk preparation, storage temperature, and the presence of seminal plasma. Accordingly, Table 2 summarizes the evidence by context rather than assigning a universal ranking to either extender category.

The evidence summarized in this table is presented according to species, preservation method, comparator, and measured outcome. Findings obtained during liquid or chilled storage should not be interpreted as evidence of cryopreservation efficacy unless freezing and thawing were evaluated directly. Likewise, numerical differences between formulations are not considered treatment effects unless supported by the statistical analysis of the cited study. References are provided separately for egg-yolk- and milk-based formulations to identify the evidence supporting each statement.

## 7. Alternatives to Conventional Animal-Derived Extenders

Although egg yolk- and milk-based extenders remain widely used, their biological variability, biosafety considerations, and species-specific limitations have stimulated evaluation of animal-origin-free and more compositionally defined systems. Current approaches include chemically defined media, plant-derived phospholipids, liposomes, membrane-modifying compounds, nanoparticles, and other biomaterials. These categories differ substantially in mechanism and level of validation and are therefore discussed separately below [108].

### 7.1. Chemically Defined Extenders

Chemically defined extenders are preservation media composed exclusively of purified and standardized constituents with known biochemical composition, thereby eliminating the variability associated with biological materials such as egg yolk and milk. These extenders typically contain defined concentrations of buffering agents, sugars, amino acids, cryoprotectants, antioxidants, and synthetic phospholipids specifically formulated to provide controlled protection during semen storage and cryopreservation. The increasing interest in chemically defined extenders is largely driven by concerns related to biosecurity, batch-to-batch variability, microbial contamination, and regulatory restrictions associated with animal-derived products [109,110].

Chemically defined extender systems have been investigated as an approach to reduce variability associated with complex biological components. Experimental evidence in goats has shown that sperm survival during liquid storage is strongly influenced by the composition of the chemically defined medium [109]. Salamon and Maxwell [111] reviewed the principal factors affecting ram semen storage, including extender composition and cryoprotective conditions, whereas Bodu et al. [112] discussed the development of more standardized extender formulations for buck semen as a strategy to improve reproducibility and reduce dependence on variable animal-derived components. These reviews provide contextual support for the potential advantages of standardized extender systems but should not be interpreted as direct comparative experimental evidence. A further advantage of chemically defined media is that their known composition facilitates mechanistic investigation of the effects of individual extender constituents on sperm function.

One of the principal advantages of chemically defined extenders is the reduction in sanitary risks associated with biological products. Animal-derived extender components may potentially harbor bacteria, fungi, viruses, endotoxins, or prion-related contaminants that compromise semen quality and biosafety. Defined formulations minimize these risks while improving quality control and extender consistency across laboratories and commercial production systems [109]. Furthermore, chemically defined extenders generally exhibit improved optical transparency, facilitating microscopic examination and computer-assisted sperm analysis (CASA) [109,110].

Amino acid supplementation has been investigated as an approach to modulate oxidative and osmotic stress during semen preservation. Compounds such as glutamine, taurine, cysteine, methionine, and proline have been evaluated under different experimental conditions, although their effects depend on species, concentration, extender composition, and preservation protocol. Bucak et al. reported that supplementation with 25 mM taurine improved post-thaw sperm motility in ram semen [113]. However, taurine did not significantly affect the hypoosmotic swelling response or post-thaw malondialdehyde, glutathione, and glutathione-peroxidase levels. Therefore, the observed improvement in motility should not be interpreted as direct evidence of enhanced membrane integrity, reduced lipid peroxidation, or mitochondrial stabilization.

Despite their advantages, chemically defined extenders may exhibit lower protective efficiency compared with conventional egg yolk systems in some species because they lack the complex lipoprotein fractions naturally present in biological products [111]. Consequently, optimization of phospholipid composition, antioxidant supplementation, and membrane stabilizers remains an active area of research aimed at improving the cryoprotective performance of chemically defined formulations.

### 7.2. Plant-Based Extenders

Plant-based extenders have been investigated as alternatives to conventional animal-derived preservation systems due to their improved biosafety, lower microbial contamination risk, greater standardization, and ethical acceptability. These extenders are primarily based on plant-derived phospholipids, particularly soy lecithin, which possesses membrane-stabilizing properties similar to those of egg yolk phospholipids. Soy lecithin contains phosphatidylcholine, phosphatidylethanolamine, and unsaturated fatty acids capable of interacting with sperm plasma membranes and reducing cold shock-induced damage during cryopreservation [114,115].

Several commercial soy lecithin-based extenders have been developed for animal semen preservation, including AndroMed^®^, Bioxcell^®^, and Ovixcell^®^. Forouzanfar et al. demonstrated that soybean lecithin could substitute for egg yolk in a Tris-based ram semen cryopreservation extender, with the best post-thaw motility and viability obtained using 1% soybean lecithin combined with 7% glycerol [116].

The mechanism of action of soy lecithin involves adsorption of phospholipid molecules onto the sperm membrane surface, thereby stabilizing membrane architecture and reducing permeability alterations induced by thermal stress. Unlike egg yolk, soy lecithin does not contain large particulate lipoprotein aggregates, resulting in greater transparency and improved compatibility with CASA systems. Furthermore, plant-based extenders eliminate the enzymatic incompatibility observed between buck seminal plasma phospholipases and egg yolk phospholipids, making them particularly attractive for goat semen cryopreservation [114,115].

In addition to soy lecithin, other plant-derived bioactive compounds have been investigated as extender supplements. Natural antioxidants extracted from green tea, rosemary, *Moringa oleifera*, *Aloe vera*, pomegranate, and curcumin have shown significant protective effects against oxidative stress during sperm preservation. For example, Susilowati et al. reported that supplementation of bull semen extenders with green tea extract significantly improved post-thaw motility, membrane integrity, and antioxidant enzyme activity [117]. A broader review of plant-derived additives has summarized experimental evidence for Moringa oleifera and other plant extracts in semen preservation across several animal species [118]. However, direct evidence in ram and buck semen remains comparatively limited, and the effects depend on plant source, extraction method, concentration, preservation protocol, and species. Therefore, further well-designed, species-specific studies are required before their routine application in ram and buck semen extenders can be recommended.

Plant-derived polysaccharides and hydrocolloids have also attracted interest due to their osmoprotective and membrane-stabilizing properties. Certain plant gums and polysaccharides may contribute to viscosity regulation and reduction in ice crystal formation during freezing. However, despite the promising results associated with plant-based extenders, inconsistencies remain among studies due to differences in lecithin concentration, extraction methods, phospholipid composition, and species-specific sperm sensitivity [118]. Therefore, further optimization and standardization are required before universal application can be achieved.

### 7.3. Synthetic and Lipid-Based Membrane Modifiers

Synthetic and lipid-based membrane modifiers are investigated to alter sperm membrane composition or provide exogenous lipid protection during cryopreservation. Their mechanisms differ from those of antioxidant nanoparticles and other nanomaterials; therefore, the categories are considered separately below [119,120].

Cholesterol-loaded cyclodextrins (CLCs) are among the best characterized membrane-modifying approaches. Cyclodextrins can deliver cholesterol to the sperm plasma membrane, increasing membrane cholesterol content and altering resistance to cold-induced phase transitions. Mocé et al. demonstrated improved cryosurvival in ram sperm treated with cholesterol-loaded cyclodextrins [120]. Effects remain dependent on dose, species, and processing protocol.

Liposomes are artificial phospholipid vesicles that may interact with sperm membranes or serve as carriers for lipids and bioactive compounds. Direct experimental evidence in ram semen includes liposome-based extender systems evaluated during refrigeration and cryopreservation, with outcomes depending on formulation, preservation method, and the sperm-quality parameter assessed [73,108]. Abbasi et al. [121] evaluated a different approach in which nanoliposomal gamma-oryzanol was added to a conventional Tris–egg yolk–glycerol cryopreservation extender using epididymal ram spermatozoa. This study should therefore be interpreted as liposomal delivery of a bioactive compound rather than replacement of animal-derived extender components by a liposome-based extender. Evidence from buffalo semen [122] is retained only as cross-species context and is not considered direct evidence for ram semen preservation. Luna-Orozco et al. [123] also provided direct experimental evidence in ram semen by comparing liposome-based and egg-yolk-based diluents during cooling and cryopreservation.

### 7.4. Nanotechnology-Based Additives

Nanotechnology-based additives should be distinguished from classical membrane stabilizers because their biological effects depend on particle composition, size, surface characteristics, aggregation state, and concentration. The potential application of nanoparticles in semen cryopreservation has been reviewed broadly [124], while direct experimental evidence in small ruminants includes selenium nanoparticles in ram semen [125,126], graphene oxide nanoparticles in Angora buck semen [127], and curcumin nanoparticles in Angora buck semen [128]. These studies primarily report laboratory sperm-quality, oxidative-status, molecular, or DNA-related endpoints, and their findings should therefore not be extrapolated automatically to field fertility or long-term reproductive safety. Where concentration- or size-dependent effects were experimentally demonstrated, these are described according to the specific nanoparticle and study conditions. In contrast, persistence in the female reproductive tract, tissue residues, long-term reproductive consequences, and environmental effects remain insufficiently investigated and should be regarded as safety questions requiring further study rather than as demonstrated adverse effects.

### 7.5. Synthetic Polymers and Other Experimental Biomaterials

Synthetic polymers can modulate ice formation and cellular cryoinjury in non-sperm cryopreservation systems [129]. However, this evidence is indirect with respect to ram and buck spermatozoa. Their potential use in small-ruminant semen extenders should therefore be regarded as experimental until direct species-specific sperm studies, safety assessments, and fertility trials are available.

Overall, membrane modifiers, liposomes, nanoparticles, and synthetic biomaterials represent mechanistically distinct approaches. Their potential benefits should be evaluated separately and interpreted according to species, preservation protocol, laboratory endpoint, fertility evidence, and safety data rather than grouped as a single class of membrane stabilizers.

## 8. Practical Applications of Semen Extenders in Small Ruminant Reproductive Biotechnology and Future Research Directions

Semen extenders have direct practical importance in small ruminant production because they determine the efficiency of artificial insemination, semen banking, genetic dissemination, disease control, and conservation of valuable or endangered breeds [2,4]. In sheep and goats, extender selection is not only a laboratory decision but also a field-level factor influencing fertility, storage duration, semen transport, and economic feasibility. Extenders are used mainly for short-term liquid storage, chilled semen preservation, and long-term cryopreservation [9]. Each application requires different priorities: liquid semen extenders must maintain sperm metabolism and motility for several hours or days, whereas cryopreservation extenders must protect spermatozoa against cold shock, osmotic stress, intracellular ice formation, oxidative damage, and post-thaw functional impairment [14,15]. Ultimately, the practical value of an extender is determined not only by its ability to preserve in vitro sperm quality but also by its capacity to achieve satisfactory fertility outcomes under field conditions. Therefore, extender selection should consider the preservation method, insemination strategy, species-specific physiology, and the intended reproductive application.

In ovine artificial insemination programs, Tris-based extenders supplemented with egg yolk and glycerol remain among the most widely used systems for ram semen cryopreservation. Their practical value is related to their ability to preserve post-thaw sperm motility, plasma membrane integrity, acrosomal status, and fertilizing capacity [56,57]. Maxwell and Salamon emphasized that extender composition, cooling rate, glycerol concentration, and thawing conditions strongly influence the fertilizing ability of frozen ram semen. In practical terms, acceptable post-thaw progressive motility in ram semen often ranges between approximately 30% and 50%, although values vary according to breed, extender, freezing protocol, and evaluation method [60]. Similarly, plasma membrane integrity frequently exceeds 50% when optimized cryopreservation protocols are applied. Fertility after cervical insemination with frozen-thawed ram semen is often lower than with fresh or chilled semen because the ovine cervix limits sperm transport; therefore, laparoscopic intrauterine insemination is frequently used to obtain higher pregnancy rates. Faigl et al. noted that artificial insemination in small ruminants can use fresh, chilled, or frozen semen, but fertility depends strongly on semen handling, insemination method, and female reproductive management [130]. Consequently, laboratory sperm quality should always be interpreted together with fertility outcomes when assessing extender performance.

In goats, practical application is more complex because buck seminal plasma contains enzymes that interact negatively with egg yolk and milk components. The egg yolk coagulating enzyme and phospholipase activity may hydrolyze extender phospholipids, producing lysophospholipids and free fatty acids that reduce sperm motility and viability. For this reason, several protocols recommend dilution soon after collection, careful control of contact time between seminal plasma and extender, or centrifugation to remove seminal plasma before freezing [131]. Leboeuf et al. highlight that buck semen extender development remains strongly influenced by the adverse effects of seminal plasma enzymes and by the need to improve post-thaw vitality and conception potential [132]. However, washing procedures must be optimized because excessive centrifugation may mechanically damage spermatozoa and increase oxidative stress. Conversely, insufficient removal of seminal plasma may allow continued phospholipase activity, resulting in further deterioration of sperm quality during cryopreservation. Therefore, optimization of seminal plasma removal remains one of the principal practical challenges in buck semen preservation.

**Figure 2:**
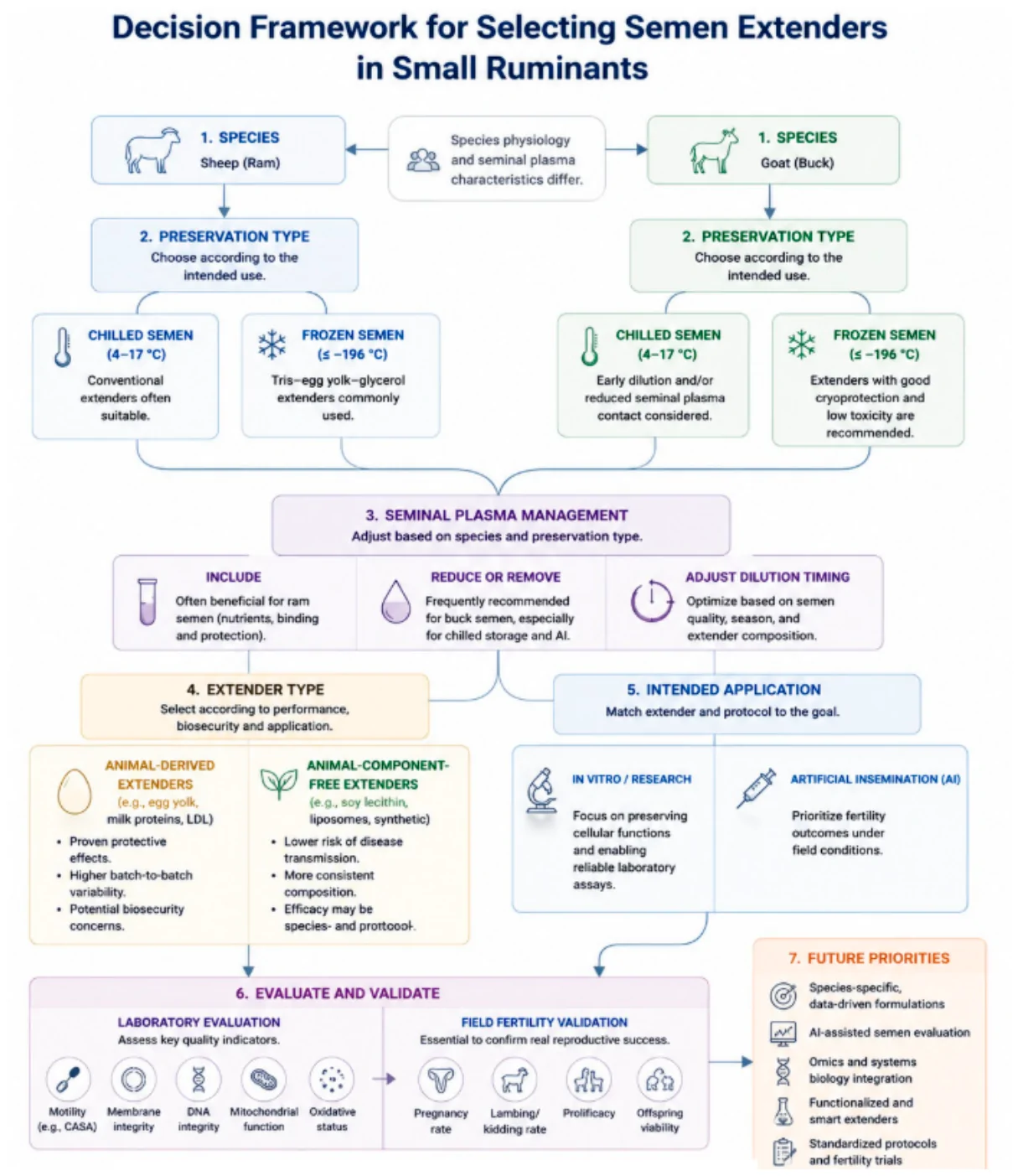
Practical applications, species-specific challenges, and future perspectives of semen extenders in small ruminant reproduction. Schematic representation of the practical applications of semen extenders in sheep and goat reproduction, highlighting the relationship between extender selection, semen preservation strategies, artificial insemination procedures, and fertility outcomes. The figure summarizes the main factors influencing cryopreservation success, including cooling and freezing protocols, oxidative stress, seminal plasma interactions, and species-specific responses of ram and buck spermatozoa. Additionally, emerging research approaches involving antioxidant supplementation, plant-based extenders, nanotechnology, and precision analytical techniques are illustrated as promising strategies for improving sperm cryosurvival, post-thaw functionality, and reproductive efficiency in small ruminants. Based on concepts discussed in the present review.

Commercial extenders have important practical relevance because they reduce laboratory variability and simplify semen processing in artificial insemination centers. Commercial extenders such as Triladyl^®^ and Optidyl^®^ (egg yolk-based formulations) and OviXcell^®^ (soy lecithin-based formulation) have been widely used in domestic animal semen preservation. However, although these extenders have been evaluated in small ruminants, their level of validation and routine application varies according to species, preservation protocol, and intended use. Egg yolk-based commercial extenders are valued for their strong cryoprotective effect, whereas soy lecithin-based extenders are increasingly used because they reduce biosecurity risks and improve standardization [114]. In addition, their standardized composition facilitates reproducible semen processing among laboratories and artificial insemination centers, reducing batch-to-batch variation associated with manually prepared extenders. Layek et al. reviewed the transition from egg yolk-based to soybean-based extenders and emphasized that plant-derived lecithin can reduce microbial contamination and batch variability while maintaining membrane-protective effects [115]. In ram semen, soybean lecithin has been evaluated as an alternative to egg yolk in Tris-based extenders. Commercial soy lecithin-based extenders, including AndroMed^®^, BioXcell^®^, and OviXcell^®^, were originally developed for specific livestock species but have also been evaluated experimentally in small-ruminant semen preservation with variable results. Mata-Campuzano et al. [133] reported that extender composition and antioxidant supplementation influenced post-thaw sperm-quality parameters. Importantly, this study evaluated laboratory endpoints and did not include an artificial-insemination fertility trial. The authors also demonstrated that the effects of antioxidant supplementation depended on the extender composition, emphasizing that interactions between antioxidants and extender formulations should be considered when optimizing cryopreservation protocols (Figure 2).

From a practical standpoint, soy lecithin extenders are particularly attractive for chilled semen transport and cryopreservation because they are clearer than egg yolk extenders, interfere less with CASA evaluation, and are compatible with biosecure semen production. Forouzanfar et al. and other authors have reported that soybean lecithin can partly or completely replace egg yolk in some ram semen protocols, although optimal concentrations differ among breeds and laboratories [116]. In buck semen, Akourki et al. reported that soybean lecithin could replace egg yolk lecithin during freezing of Bengal buck semen and also reduced sperm agglutination problems [134]. Nevertheless, not all studies show complete equivalence between soy lecithin and egg yolk, and some reports indicate that egg yolk may still better preserve viability, acrosomal integrity, or membrane functionality under specific conditions.

Another important practical application is the incorporation of antioxidants into extenders. During chilled storage and cryopreservation, spermatozoa are exposed to reactive oxygen species, which promote lipid peroxidation, mitochondrial dysfunction, DNA damage, and loss of motility. Therefore, antioxidant supplementation is increasingly used to improve extender performance [11]. Compounds such as glutathione, cysteine, taurine, melatonin, vitamin E, vitamin C, resveratrol, catalase, selenium nanoparticles, and plant extracts have been evaluated in ram and buck semen [11]. Primary experimental studies have reported compound- and protocol-dependent effects on sperm motility, membrane integrity, and oxidative status, including studies evaluating thymoquinone, taurine, cysteine/glutathione combinations, and selenium nanoparticles [70,104,113,125,126]. These findings indicate that antioxidant supplementation may be beneficial under specific conditions, but requires optimization according to concentration, extender composition, and preservation protocol.

In field conditions, the choice of extender must be adapted to the insemination strategy. Fresh and chilled semen are generally more suitable for short-distance use and may produce better fertility than frozen-thawed semen when insemination is performed cervically [130,135,136]. Frozen semen is more useful for long-distance transport, genetic exchange, conservation programs, and international germplasm movement, but requires stricter control of extender formulation, sperm concentration, cooling curve, equilibration time, packaging system, and thawing temperature [5]. For ram semen, frozen-thawed doses are commonly used with laparoscopic intrauterine insemination to bypass the cervical barrier, whereas goats may show better cervical insemination outcomes depending on semen quality, extender, and timing of insemination. Thus, extender development should not be evaluated only by in vitro sperm parameters; although improvements in motility, viability, membrane integrity, mitochondrial activity, and oxidative status are essential indicators of extender performance, pregnancy rate, lambing or kidding rate, prolificacy, and offspring viability remain the most relevant criteria for validating extender efficiency under commercial production conditions [135,136].

Representative studies that included in vivo reproductive outcomes are summarized in Table 3. This focused synthesis is intended to distinguish field validation from studies reporting only post-thaw laboratory parameters; it is not a systematic or PRISMA-style evidence extraction.

The available fertility evidence indicates that reproductive outcomes cannot be attributed to extender composition independently of species, semen source, preservation method, insemination route, sperm dose, and female reproductive management. In several studies, procedural factors varied together with extender-related variables, limiting the extent to which an independent extender effect can be inferred. Moreover, the limited number and heterogeneity of in vivo studies emphasize the need to distinguish numerical differences from statistically demonstrated treatment effects and laboratory sperm-quality improvements from reproductive benefits under field conditions.

Future research should focus on developing species-specific and functionally targeted extenders rather than universal formulations. Ram and buck spermatozoa differ in membrane characteristics, seminal-plasma interactions, cryotolerance, and responses to animal-derived and plant-derived extender components. Therefore, future studies should compare extender formulations under standardized conditions using adequate numbers of males, multiple breeds where possible, and reproductive endpoints. A major limitation of the current literature is that many studies report post-thaw motility, viability, membrane integrity, or oxidative-status parameters, whereas comparatively few include pregnancy or lambing/kidding outcomes. Because improvements in in vitro sperm quality do not necessarily translate into improved fertility, future experiments should integrate laboratory assessments with adequately designed in vivo reproductive trials.

A second research priority is the refinement of animal-origin-free extender systems. Soy lecithin, synthetic phospholipids, liposomes, cholesterol-loaded cyclodextrins, and chemically defined media may improve standardization and reduce dependence on biological materials. However, their efficacy depends on concentration, phospholipid profile, extender osmolarity, cryoprotectant compatibility, and species-specific sperm characteristics. Future studies should determine whether these formulations can consistently reproduce laboratory and fertility outcomes obtained with conventional extenders under standardized experimental conditions.

A third research direction involves the use of molecular approaches to better characterize sperm cryotolerance. Omics studies have already provided direct mechanistic information in small ruminants. In ram spermatozoa, proteomic and phosphoproteomic analyses have identified cryopreservation-associated alterations in proteins and signaling pathways [8,79], while integrated transcriptomic, metabolomic, and proteomic analyses have been applied to buck semen cryopreservation [10]. These approaches can identify molecular changes associated with membrane stability, metabolism, oxidative stress, and post-thaw sperm function and may provide candidate biomarkers or biological targets for future extender optimization. However, current omics studies primarily characterize molecular responses to cryopreservation and do not yet demonstrate that omics-guided extender formulation improves fertility in ram or buck semen. Nanotechnology represents a related but distinct experimental approach. Nanoparticle- and nanocarrier-based strategies may modify antioxidant delivery or sperm protection, but their effects are compound-, size-, concentration-, and protocol-dependent. Although experimental studies have reported improvements in selected sperm-quality endpoints, dose–response effects, reproductive safety, residues, and field fertility require further evaluation before routine application [137].

Another emerging area of interest is seminal-plasma extracellular vesicles, which have been implicated in sperm maturation, intercellular communication, and membrane-related processes. Current veterinary literature supports their biological relevance, and cryopreservation and extender composition have been shown to influence extracellular vesicles in bull semen [138,139]. However, these studies do not demonstrate that supplementation of semen extenders with extracellular vesicles improves sperm preservation in small ruminants. Therefore, extracellular-vesicle supplementation should currently be regarded as a research hypothesis rather than an established preservation strategy.

Artificial-intelligence and machine-learning approaches represent an additional future direction. Their demonstrated application in animal semen research currently relates primarily to automated semen assessment rather than to extender formulation. For example, an artificial-intelligence system based on convolutional neural networks has been evaluated for automated bull sperm morphology classification, achieving an overall accuracy of 82% and a precision of 85% [140]. Such approaches may improve standardization and reduce operator dependence in semen evaluation. However, direct evidence that artificial intelligence can optimize extender composition, predict individual cryotolerance, or improve fertility in ram or buck semen remains limited. Therefore, integration of AI with CASA, flow cytometry, fertility data, and omics-derived biomarkers should presently be considered a prospective research direction rather than an established component of small-ruminant semen preservation.

## 9. Conclusions

Semen extenders remain a fundamental component of assisted reproductive technologies in small ruminants by protecting spermatozoa against cold shock, osmotic imbalance, oxidative stress, and cryoinjury during chilled preservation and cryopreservation. Among currently available preservation systems, egg yolk-based extenders remain the most extensively validated and widely used option, particularly for ram semen cryopreservation, owing to their consistent membrane-protective effects and the substantial body of supporting evidence. In contrast, buck semen requires species-specific preservation strategies because of the interaction between seminal plasma enzymes and animal-derived extender components, which has stimulated the development of alternative formulations.

Plant-based extenders, chemically defined formulations, antioxidants, membrane stabilizers, and nanoparticle-based approaches have demonstrated encouraging results, primarily through improvements in laboratory sperm-quality parameters such as motility, membrane integrity, mitochondrial activity, and oxidative status. However, evidence demonstrating consistent improvements in fertility under field conditions remains limited, and laboratory outcomes should not be interpreted as direct indicators of reproductive performance. Although several emerging technologies, including nanotechnology-, omics-, and extracellular vesicle-based approaches, offer promising opportunities for future research, their application in small-ruminant semen preservation remains largely experimental, and important questions regarding standardization, biosafety, optimal concentrations, long-term safety, and reproducibility remain to be resolved.

Overall, the available evidence indicates that no universally optimal semen extender is likely to be applicable across all small-ruminant species or preservation protocols, as extender performance depends on species-specific sperm physiology, seminal plasma composition, preservation method, and processing conditions. Future research should therefore focus on developing species-specific, evidence-based, and well-standardized extender formulations, with greater emphasis on well-designed fertility trials to validate whether improvements in laboratory sperm-quality parameters translate into meaningful reproductive benefits under commercial conditions.

## Figures and Tables

**Table 1:** Major conventional components of semen extenders and their biological functions in small ruminant semen preservation.

Extender Component	Representative Examples	Primary Biological Function	Mechanism of Action	Main Advantages	Main Limitations	References
Buffers	Tris, sodium citrate, phosphate	Maintenance of pH and osmotic balance	Stabilize extracellular pH, ionic equilibrium, and osmolarity during preservation	Contribute to pH stability and osmotic balance	Buffer performance varies according to formulation and storage conditions	[56,57,58,59]
Energy substrates	Fructose, glucose, lactose	ATP production and metabolic support	Provide substrates for sperm metabolism and contribute to osmotic regulation	Maintain motility and membrane function	Excessive metabolism may increase ROS production	[60,61,62]
Penetrating cryoprotectants	Glycerol, DMSO, ethylene glycol	Protection against intracellular ice formation	Replace intracellular water and reduce freezing injury	Effective intracellular cryoprotection	Cytotoxicity and osmotic stress at excessive concentrations; species-dependent responses	[63,64,65,66]
Non-penetrating cryoprotectants	Trehalose, sucrose	Osmotic protection and membrane stabilization	Promote controlled dehydration and stabilize membrane phospholipids	Reduce osmotic stress and improve membrane integrity	Effectiveness depends on concentration and freezing protocol	[67,68]
Antioxidants	Melatonin, glutathione, vitamin E, catalase, plant extracts	Reduction in oxidative stress	Neutralize excessive ROS and preserve membrane integrity	May improve post-thaw sperm quality under optimized conditions	Dose-dependent effects; excessive supplementation may disrupt physiological redox balance	[11,37,70]
Membrane-protective components	Egg yolk, LDL, milk proteins, soy lecithin	Plasma membrane stabilization	Supply phospholipids and cholesterol to preserve membrane fluidity	Protect against cold shock and cryoinjury	Animal-derived products may interact with buck seminal plasma enzymes; plant-based alternatives may vary in efficacy	[71,72,73,74]
Antimicrobial agents	Penicillin–streptomycin, gentamicin, lincomycin–spectinomycin	Control of bacterial contamination	Inhibit bacterial growth during semen storage	Reduce bacterial proliferation during semen storage	Potential sperm toxicity and antimicrobial resistance concerns	[53,54,55]

**Table 2:** Comparative analysis between egg yolk-based and milk-based extenders used in small ruminant semen preservation.

Parameter/Experimental Context	Egg-Yolk-Based Extenders	References	Milk-Based Extenders	References
Principal protective components	Egg-yolk phospholipids, cholesterol, and low-density lipoprotein fractions contribute to sperm-membrane protection during cooling and freezing.	[42,72,73,74]	Milk proteins and casein-containing fractions contribute to sperm protection, with effects depending on milk preparation and the complete extender formulation.	[42,90,94]
Ram semen—liquid/chilled preservation	Egg-yolk-containing formulations have been evaluated for preservation of ram semen during refrigerated storage; outcomes depend on extender composition, storage temperature, and duration.	[57,75,90]	Milk-based formulations have been evaluated directly for liquid/chilled ram semen and have maintained sperm quality under defined storage protocols.	[90,92,94,95]
Ram semen—cryopreservation	Tris–egg yolk and LDL-containing formulations have been evaluated directly for ram semen cryopreservation, with effects reported for post-thaw motility, membrane/acrosomal integrity, and other sperm-quality endpoints.	[71,72,77,80,85]	Milk-containing or milk-derived formulations have been evaluated in selected ram cryopreservation protocols, but results from liquid-storage studies should not be extrapolated directly to frozen-thawed semen.	[72]
Ram semen—in vivo fertility evidence	Fertility outcomes have been reported following AI with ram semen preserved in egg-yolk-containing formulations; interpretation depends on preservation conditions and insemination procedure.	[71,75]	Field studies have evaluated fertility following AI with liquid/chilled ram semen preserved in milk-based formulations.	[92,94,95]
Buck semen—interaction with seminal plasma	In egg-yolk-containing media, seminal-plasma enzymatic activity can hydrolyze egg-yolk phospholipids, with formation of lysophospholipids and free fatty acids.	[39,81,82,83]	In milk-based media, BUSgp60/GoPLRP2 hydrolyzes residual milk triglycerides and releases free fatty acids, particularly oleic acid, which can impair sperm motility and viability.	[40,81]
Buck semen—cryopreservation and fertility evidence	Egg-yolk-containing extenders have been evaluated directly for buck semen cryopreservation using post-thaw sperm-quality endpoints, while reproductive outcomes following AI have been reported in comparative studies.	[89,96]	Skim-milk-based extenders have been evaluated directly for buck semen cryopreservation using post-thaw sperm-quality and ultrastructural endpoints, while reproductive outcomes following AI have been reported in comparative studies.	[96,97]
Microbial contamination/biosecurity	Because egg yolk is a biological material, hygienic sourcing, preparation, and semen-processing procedures remain important. No universal contamination ranking is assigned in this review.	[42,53,54,55]	Similar microbiological considerations apply to milk-derived systems and depend on source, processing, handling, and antimicrobial strategy; no universal contamination ranking is assigned.	[42,53,54,55]
Interpretative limitation	Reported biological effects are protocol-specific and should be interpreted according to species, preservation method, formulation, comparator, and measured endpoint.	[42]	Reported biological effects are protocol-specific and should be interpreted according to species, preservation method, formulation, comparator, and measured endpoint.	[42]

**Table 3:** Representative studies reporting in vivo reproductive outcomes following the use of semen extenders or extender-related preservation strategies in small ruminants. AI = artificial insemination; UHT = ultra-high-temperature-treated milk; INRA 96^®^ = commercial milk-protein-based semen extender; Tris = tris(hydroxymethyl)aminomethane. Numerical differences are described as treatment effects only when supported by the statistical analysis reported in the original study. Where insemination route, semen condition, or other procedural factors differed among groups, these factors are identified because they may prevent isolation of the extender effect.

Study	Species, Semen, and Females Inseminated	Extender/Preservation Context	AI Route and Reproductive Endpoint	Main Fertility Result/Statistical Interpretation	Principal Limitation
Berean et al. [5]	Lacaune ram; frozen-thawed ejaculated semen Females inseminated: Control: 9; L-arginine: 10; eugenol: 10; five additional ewes were excluded because transcervical passage was not possible	Biladyl-based cryopreservation extender; control vs. L-arginine or eugenol supplementation	Transcervical AI; pregnancy diagnosed by transabdominal ultrasonography 75 days after AI	Pregnancy occurred in 5/9 (55.6%), 6/10 (60.0%), and 8/10 (80.0%) ewes in the control, L-arginine, and eugenol groups, respectively. Numerical differences should be interpreted according to the statistical analysis reported in the original study.	Small treatment groups; five ewes were excluded because transcervical passage could not be achieved.
Paulenz et al. [92]	Norwegian Crossbred ram; cooled liquid ejaculated semen Females inseminated: 561 ewes in total	Milk-based vs. Tris-based extender; split-sample design	Single or double vaginal AI in natural estrus; 25-day non-return and lambing rates	The milk-based extender resulted in approximately 10 percentage points higher 25-day non-return and lambing rates than the Tris-based extender (*p* < 0.01).	Fertility was influenced by both extender type and single vs. double insemination; the extender effect was therefore evaluated together with insemination strategy.
Olivera-Muzante et al. [94]	Merino ram; fresh or chilled ejaculated semen Females inseminated: 1198 ewes across two field experiments	UHT skim milk or INRA 96^®^ containing 5% egg yolk; ±2% glycerol; storage for 24 or 48 h	Cervical AI in spontaneous estrus; conception determined by ultrasonography 40 days after AI	INRA 96^®^- and UHT-based systems produced similar conception rates (56.7% vs. 55.4%; *p* > 0.05). Addition of glycerol and extension of storage from 24 to 48 h did not produce significant differences in the comparisons reported.	Several extender formulations and storage durations were evaluated across two field experiments rather than in a single isolated extender comparison.
Gogol et al. [71]	Wrzosówka ram; frozen-thawed ejaculated semen Females inseminated: Not reported	Milk- or Tris-based extender supplemented with egg yolk or soybean lecithin	AI; lambing rate	The milk–soybean lecithin formulation produced a numerically higher lambing rate than Tris–egg yolk (42.9% vs. 16.7%), but the difference was not statistically significant.	The numerical fertility difference was not statistically demonstrated and should not be interpreted as evidence of superiority.
Liang et al. [96]	Yunshang Black buck; frozen-thawed ejaculated semen Females inseminated: Not reported	AndroMed^®^, Optidyl^®^, soybean phosphatidylcholine, or skim-milk extender	Exocervical AI; pregnancy, lambing, and twinning rates	Pregnancy and lambing rates were significantly lower in the skim-milk group than in the other extender groups (*p* < 0.05), whereas AndroMed^®^, Optidyl^®^, and soybean-phosphatidylcholine groups did not differ significantly (*p* > 0.05). Twinning rates were similar among groups.	Several complete extender formulations were compared; therefore, the effect cannot be attributed to a single extender constituent.
Abbasi et al. [121]	Shall ram; frozen-thawed epididymal spermatozoa Females inseminated: 140 synchronized ewes randomly allocated among seven treatment groups	Conventional Tris–egg yolk–glycerol extender supplemented with free gamma-oryzanol or nanoliposomal gamma-oryzanol	Laparoscopic intrauterine AI; 100 × 10^6^ spermatozoa per 0.25 mL straw; pregnancy by ultrasonography, with birth, lambing, and twinning outcomes also assessed	The 100 µM nanoliposomal gamma-oryzanol group showed the highest pregnancy rate (79.16%), together with significantly improved fertility outcomes compared with the control (*p* < 0.05).	Epididymal spermatozoa were used, and the nanoliposome was an additive to a conventional egg-yolk/glycerol extender rather than a replacement liposome-based extender.
Hiwasa et al. [136]	Suffolk and Suffolk-crossbred sheep; fresh-diluted or frozen-thawed semen Females inseminated: 80 ewes in total; 54 included in the pregnancy experiment	Synthetic AndroMed^®^ extender; fresh-diluted or frozen-thawed semen	Cervical vs. laparoscopic intrauterine AI; fertilization and pregnancy outcomes	Intrauterine AI with frozen-thawed semen produced a 72.2% pregnancy rate, compared with 5.5% after cervical AI with fresh-diluted semen and 0% after cervical AI with frozen-thawed semen (*p* < 0.05).	The principal comparison concerned insemination route and semen condition rather than extender composition; therefore, an independent extender effect cannot be inferred.

## Data Availability

No new data were created or analyzed in this study. Data sharing is not applicable to this article.

## References

[1] Sargison N.D. (2020). The critical importance of planned small ruminant livestock health and production in addressing global challenges surrounding food production and poverty alleviation. N. Z. Vet. J..

[2] Dumont B., Groot J.C.J., Tichit M. (2018). Review: Make ruminants green again-how can sustainable intensification and agroecology converge for a better future?. Animal.

[3] Pretty J., Bharucha Z.P. (2014). Sustainable intensification in agricultural systems. Ann. Bot..

[4] Lalljee S.V., Soundararajan C., Singh Y.D., Sargison N.D. (2019). The potential of small ruminant farming as a means of poverty alleviation in rural southern India. Trop. Anim. Health Prod..

[5] Berean D.I., Blaga-Petrean A., Bogdan I., Bogdan S., Tamas-Krumpe O.M., Cimpean R., Pall E., Nap M.E., Bogdan L.M. (2022). Effect of L-arginine and Eugenol on Ram Semen Kinematic Parameters and Post Thawed Fertility Rate after Trans-cervical Artificial Insemination. Indian J. Anim. Res..

[6] Cofré E., Peralta O.A., Raggi A., De Los Reyes M., Sales F., González-Bulnes A., Parraguez V.H. (2018). Ram semen deterioration by short-term exposure to high altitude is prevented by improvement of antioxidant status. Animal.

[7] Ahmadi M.H.N., Mirzaei A., Divar M.R., Derafshani F. (2025). Optimising Filtered Egg Yolk Extenders for Long-Term Cold Preservation of Ram Sperm: Effects of Osmolarity and Egg Yolk Concentration. Reprod. Domest. Anim..

[8] Zang S., Yang X., Ye J., Mo X., Zhou G., Fang Y. (2024). Quantitative phosphoproteomics explain cryopreservation-induced reductions in ram sperm motility. J. Proteom..

[9] Hidalgo M., Rodríguez I., Dorado J.M. (2007). The effect of cryopreservation on sperm head morphometry in Florida male goat related to sperm freezability. Anim. Reprod. Sci..

[10] Zhong T., Wu S., Chen G., Zhan S., Wang L., Cao J., Guo J., Li L., Zhang H., Niu L. (2025). Integrated analyses of transcriptomes, metabolomes, and proteomes unveil the role of FoXO signaling axis in buck semen cryopreservation. Theriogenology.

[11] Berean D.I., Bogdan L.M., Cimpean R. (2024). Advancements in Understanding and Enhancing Antioxidant-Mediated Sperm Cryopreservation in Small Ruminants: Challenges and Perspectives. Antioxidants.

[12] Yánez-Ortiz I., Catalán J., Rodríguez-Gil J.E., Miró J., Yeste M. (2022). Advances in sperm cryopreservation in farm animals: Cattle, horse, pig and sheep. Anim. Reprod. Sci..

[13] Andrade A.F.C., Knox R.V., Torres M.A., Pavaneli A.P.P. (2022). What is the relevance of seminal plasma from a functional and preservation perspective?. Anim. Reprod. Sci..

[14] Araújo Silva R.A.J., Monteiro M.M., Costa J.A.S., Mergulhão F.C.C., Monteiro P.L.J., Silva D.M.F., Santos Filho A.S., Souza A.F., Batista A.M., Guerra M.M.P. (2022). Dimethylformamide Preserves the Integrity of Cryopreserved Goat Semen in a Soybean Lecithin-Based Extender. Biopreserv. Biobank..

[15] Najafi A., Daghigh-Kia H., Dodaran H.V., Mehdipour M., Alvarez-Rodriguez M. (2017). Ethylene glycol, but not DMSO, could replace glycerol inclusion in soybean lecithin-based extenders in ram sperm cryopreservation. Anim. Reprod. Sci..

[16] Akhtar M.F., Ma Q., Li Y., Chai W., Zhang Z., Li L., Wang C. (2022). Effect of Sperm Cryopreservation in Farm Animals Using Nanotechnology. Animals.

[17] Salmon V.M., Leclerc P., Bailey J.L. (2017). Novel technical strategies to optimize cryopreservation of goat semen using cholesterol-loaded cyclodextrin. Cryobiology.

[18] Curry M.R., Watson P.F. (1994). Osmotic effects on ram and human sperm membranes in relation to thawing injury. Cryobiology.

[19] Miyata H., Shimada K., Kaneda Y., Ikawa M. (2024). Development of functional spermatozoa in mammalian spermiogenesis. Development.

[20] López-González I., Oseguera-López I., Castillo R., Darszon A. (2024). Influence of extracellular ATP on mammalian sperm physiology. Reprod. Fertil. Dev..

[21] Bellezza I., Minelli A. (2017). Adenosine in sperm physiology. Mol. Asp. Med..

[22] Güngör İ.H., Türk G., Dayan Cinkara S., Acisu T.C., Tektemur A., Yeni D., Avdatek F., Arkali G., Koca R.H., Özer Kaya Ş. (2024). Reduction of cryopreservation-induced structural, functional and molecular damages in ram sperm by hydrated C_60_ fullerene. Reprod. Domest. Anim..

[23] Emamverdi M., Zhandi M., Zare Shahneh A., Sharafi M., Akbari-Sharif A. (2013). Optimization of ram semen cryopreservation using a chemically defined soybean lecithin-based extender. Reprod. Domest. Anim..

[24] White I.G. (1993). Lipids and calcium uptake of sperm in relation to cold shock and preservation: A review. Reprod. Fertil. Dev..

[25] Pérez-Pé R., Cebrián-Pérez J.A., Muiño-Blanco T. (2001). Semen plasma proteins prevent cold-shock membrane damage to ram spermatozoa. Theriogenology.

[26] García-López N., Ollero M., Cebrián-Pérez J.A., Muiño-Blanco T. (1996). Reversion of thermic-shock effect on ram spermatozoa by adsorption of seminal plasma proteins revealed by partition in aqueous two-phase systems. J. Chromatogr. B Biomed. Appl..

[27] Ollero M., Cebrian-Perez J.A., Muiño-Blanco T. (1997). Improvement of cryopreserved ram sperm heterogeneity and viability by addition of seminal plasma. J. Androl..

[28] Hamilton T.R., de Castro L.S., Delgado J.d.C., de Assis P.M., Siqueira A.F., Mendes C.M., Goissis M.D., Muiño-Blanco T., Cebrián-Pérez J.Á., Nichi M. (2016). Induced lipid peroxidation in ram sperm: Semen profile, DNA fragmentation and antioxidant status. Reproduction.

[29] Partyka A., Łukaszewicz E., Niżański W. (2012). Effect of cryopreservation on sperm parameters, lipid peroxidation and antioxidant enzymes activity in fowl semen. Theriogenology.

[30] Coyan K., Başpınar N., Bucak M.N., Akalın P.P., Ataman M.B., Omür A.D., Güngör S., Küçükgünay S., Ozkalp B., Sarıözkan S. (2010). Influence of methionine and dithioerythritol on sperm motility, lipid peroxidation and antioxidant capacities during liquid storage of ram semen. Res. Vet. Sci..

[31] Varisli O., Bozkaya F., Aydilek N., Taskin A. (2022). Relationship between toxicity of cryoprotectants, osmotic and oxidative stresses in awassi ram sperm. Cryo Lett..

[32] Nur Z., Zik B., Ustuner B., Sagirkaya H., Ozguden C.G. (2010). Effects of different cryoprotective agents on ram sperm morphology and DNAintegrity. Theriogenology.

[33] Fraser L., Strzeżek J., Kordan W. (2011). Effect of freezing on sperm nuclear DNA. Reprod. Domest. Anim..

[34] Van de Hoek M., Rickard J.P., de Graaf S.P. (2022). Motility Assessment of Ram Spermatozoa. Biology.

[35] Zhang L., Sun Y., Jiang C., Sohail T., Sun X., Wang J., Li Y. (2024). Damage to Mitochondria During the Cryopreservation, Causing ROS Leakage, Leading to Oxidative Stress and Decreased Quality of Ram Sperm. Reprod. Domest. Anim..

[36] Slowinska M., Liszewska E., Judycka S., Konopka M., Ciereszko A. (2018). Mitochondrial membrane potential and reactive oxygen species in liquid stored and cryopreserved turkey (*Meleagris gallopavo*) spermatozoa. Poult. Sci..

[37] Santiani A., Evangelista S., Sepúlveda N., Risopatrón J., Villegas J., Sánchez R. (2014). Addition of superoxide dismutase mimics during cooling process prevents oxidative stress and improves semen quality parameters in frozen/thawed ram spermatozoa. Theriogenology.

[38] O’ Meara C.M., Hanrahan J.P., Prathalingam N.S., Owen J.S., Donovan A., Fair S., Ward F., Wade M., Evans A.C., Lonergan P. (2008). Relationship between in vitro sperm functional tests and in vivo fertility of rams following cervical artificial insemination of ewes with frozen-thawed semen. Theriogenology.

[39] Purdy P.H. (2006). A Review on Goat Sperm Cryopreservation. Small Rumin. Res..

[40] Pellicer-Rubio M.T., Magallon T., Combarnous Y. (1997). Deterioration of goat sperm viability in milk extenders is due to a bulbourethral 60-kilodalton glycoprotein with triglyceride lipase activity. Biol. Reprod..

[41] Premrov Bajuk B., Pihlar T., Pogačnik N., Klinc P. (2018). Dialysis of the Goat Semen and Its Effect on the Quality of Frozen/Thawed Spermatozoa Processed in the Presence of Egg Yolk. Anim. Reprod. Sci..

[42] Bustani G.S., Baiee F.H. (2021). Semen extenders: An evaluative overview of preservative mechanisms of semen and semen extenders. Vet. World.

[43] Halawa W., Khnissi S., Bensouf I., Bejaoui B., Chalouati H., Salman M., M’Hamdi N. (2025). Impact of semen extenders, storage duration, and insemination timing on semen quality and reproductive performance in Palestinian Assaf sheep. Vet. World.

[44] Dziekońska A., Neuman N.M., Burdal K.K., Wiszniewska-Łaszczych A., Bogdaszewski M. (2022). The Effect of Different Extenders on the Quality Characteristics of European Red Deer Epididymal Sperm Stored at 5 °C. Animals.

[45] Yamashiro H., Kumamoto K., Wang H., Yamashita Y., Terada T. (2006). Effect of semen collection in extender solution on the characteristics of goat spermatozoa. J. Reprod. Dev..

[46] Zhao B.T., Han D., Xu C.L., Luo M.J., Chang Z.L., Tan J.H. (2009). Protocol optimization for long-term liquid storage of goat semen in a chemically defined extender. Reprod. Domest. Anim..

[47] Barbas J.P., Mascarenhas R.D. (2009). Cryopreservation of domestic animal sperm cells. Cell Tissue Bank..

[48] Rodriguez-Gil J.E. (2006). Mammalian sperm energy resources management and survival during conservation in refrigeration. Reprod. Domest. Anim..

[49] Anel L., Alvarez M., Martinez-Pastor F., Garcia-Macias V., Anel E., de Paz P. (2006). Improvement strategies in ovine artificial insemination. Reprod. Domest. Anim..

[50] Hameed N., Akhter S., Souza-Fabjan J.M.G., Zubair M., Irfan-Ur-Rehman Khan M. (2024). Effects of different extenders, storage temperatures, and antioxidant supplementation on chilled semen quality: A review. Trop. Anim. Health Prod..

[51] Xue S.H., Xu B.B., Yan X.C., Zhang J.X., Su R. (2025). Sperm Membrane Stability: In-Depth Analysis from Structural Basis to Functional Regulation. Vet. Sci..

[52] Sohail T., Tharwat M., Shaukat A., Nassar N., Chen F., Sun X., Alshanbari F.A., Li Y. (2026). Liquid Storage and Cryopreservation of Ram Semen: Storage-Associated Damage and the Role of Non-Enzymatic Antioxidants. Vet. Sci..

[53] Morrell J.M., Cojkic A., Malaluang P., Ntallaris T., Lindahl J., Hansson I. (2024). Antibiotics in semen extenders—A multiplicity of paradoxes. Reprod. Fertil. Dev..

[54] Schulze M., Nitsche-Melkus E., Hensel B., Jung M., Jakop U. (2020). Antibiotics and their alternatives in Artificial Breeding in livestock. Anim. Reprod. Sci..

[55] Garriga F., Bonet S., Yeste M. (2025). Assessment of Bacterial Contamination in Semen. Methods Mol. Biol..

[56] Graham E.F., Crabo B.G., Pace M.M. (1978). Current status of semen preservation in the ram, boar and stallion. J. Anim. Sci..

[57] López A., Söderquist L., Rodriguez-Martinez H. (1999). Sperm viability in ram semen diluted and stored in three different extenders. Acta Vet. Scand..

[58] Paul R.K., Balaganur K., Bahire S.V., Kumar D., Singh R. (2018). Supplementation of cauda epididymal plasma improves sperm characteristics following liquid preservation of ram semen at 3–5 °C. Reprod. Fertil. Dev..

[59] Akhter S., Zubair M., Mahmood M., Andrabi S.M.H., Hameed N., Ahmad E., Saleemi M.K. (2023). Effects of vitamins C and E in tris citric acid glucose extender on chilled semen quality of Kail ram during different storage times. Sci. Rep..

[60] Maxwell W.M., Salamon S. (1993). Liquid storage of ram semen: A review. Reprod. Fertil. Dev..

[61] Ben Moula A., Hamidallah N., Badi A., El Fadili M., El Amiri B. (2024). Adjusting ram semen preservation: Exploring the impact of oxygen exposure during liquid storage. Reprod. Domest. Anim..

[62] Del Valle I., Mendoza N., Casao A., Cebrián-Pérez J.A., Pérez-Pé R., Muiño-Blanco T. (2010). Significance of non-conventional parameters in the evaluation of cooling-induced damage to ram spermatozoa diluted in three different media. Reprod. Domest. Anim..

[63] Paul R.K., Kumar D., Singh R. (2021). Carboxymethyl cellulose and glycerol act synergistically as cryoprotectant during cryopreservation of ram semen. Cryobiology.

[64] Saha A., Asaduzzaman M., Bari F.Y. (2022). Cryopreservation Techniques for Ram Sperm. Vet. Med. Int..

[65] Saberivand A., Pashapour S., Noghani A.E., Namvar Z. (2022). Synergistic effect of royal jelly in combination with glycerol and dimethyl sulfoxide on cryoprotection of Romanov ram sperm. Cryobiology.

[66] Silva E.C., Cajueiro J.F., Silva S.V., Vidal A.H., Soares P.C., Guerra M.M. (2012). In vitro evaluation of ram sperm frozen with glycerol, ethylene glycol or acetamide. Anim. Reprod. Sci..

[67] Öztürk A.E., Bodu M., Bucak M.N., Ağır V., Özcan A., Keskin N., İli P., Topraggaleh T.R., Sidal H., Başpınar N. (2020). The synergistic effect of trehalose and low concentrations of cryoprotectants can improve post-thaw ram sperm parameters. Cryobiology.

[68] Arando A., Gonzalez A., Delgado J.V., Arrebola F.A., Perez-Marín C.C. (2017). Storage temperature and sucrose concentrations affect ram sperm quality after vitrification. Anim. Reprod. Sci..

[69] Keskin N., Erdogan C., Bucak M.N., Ozturk A.E., Bodu M., Ili P., Baspinar N., Dursun S. (2020). Cryopreservation Effects on Ram Sperm Ultrastructure. Biopreserv. Biobank..

[70] İnanç M.E., Güngör Ş., Avdatek F., Yeni D., Gülhan M.F., Olğaç K.T., Denk B., Taşdemir U. (2022). Thymoquinone improves motility, plasma membrane integrity and DNA integrity of frozen-thawed ram semen. Andrologia.

[71] Gogol P., Bryła M., Trzcińska M., Bochenek M. (2019). Quality parameters and fertility of ram semen cryopreserved in egg yolk and soybean lecithin supplemented extenders. Pol. J. Vet. Sci..

[72] Bahmani S., Eslami M., Farrokhi-Ardabili F., Imani M., Batavani R.A. (2023). Evaluation of Chicken Egg Yolk Plasma and Low-Density Lipoprotein Alone or Enriched with Ewe or Cow Skim Milk in Tris-Citric Acid-Based Diluent for Cryostorage of Ram Semen. Biopreserv. Biobank..

[73] de Almeida R.A., da Silva L.S., de Lima L.G.F., Lourencetti F.A., Freitas-Dell Aqua C., Ferreira J.C.P., Oba E. (2025). Egg-yolk- and a liposome-based extenders: Refrigeration time and effects on ram semen quality. Anim. Reprod..

[74] Al Ahmad M.Z., Chatagnon G., Amirat-Briand L., Moussa M., Tainturier D., Anton M., Fieni F. (2008). Use of glutamine and low density lipoproteins isolated from egg yolk to improve buck semen freezing. Reprod. Domest. Anim..

[75] Arando A., Delgado J.V., León J.M., Nogales S., Navas-González F.J., Pizarro M.G., Pérez-Marín C.C. (2019). Effect of Three Commercial Extenders on Sperm Motility and Fertility in Liquid Ram Semen Stored at 15 °C or 5 °C. Acta Vet. Hung..

[76] Mujitaba M.A., Kútvölgyi G., Radnai Szentpáli J., Debnár V.J., Tokár A., Vass N., Bodó S. (2024). The Influence of Three Commercial Soy Lecithin-Based Semen Extenders and Two Spermatozoa Concentrations on the Quality of Pre-Freeze and Post-Thaw Ram Epididymal Spermatozoa. Animals.

[77] Khatun A., Fazili M.R., Malik A.A., Shah R.A., Khan H.M., Choudhury A.R., Naikoo M., Lone F.A., Malik A. (2021). In vitro Assessment of Tris Egg Yolk and Soybean Lecithin Based Extenders for Cryopreservation of Crossbred Ram Semen. Cryo Lett..

[78] Saratsi A., Samartzi F., Tsiokos D., Theodosiadou E.K., Panagiotidis I., Ligda C., Rekkas C.A. (2024). Effect of Three Commercially Available Extenders Containing Phospholipids of Different Sources on Skopelos Buck Liquid-Stored Sperm Quality. Vet. Sci..

[79] Pini T., Rickard J.P., Leahy T., Crossett B., Druart X., de Graaf S.P. (2018). Cryopreservation and egg yolk medium alter the proteome of ram spermatozoa. J. Proteom..

[80] Alcay S., Berk Toker M., Gokce E., Ustuner B., Tekin Onder N., Sagirkaya H., Nur Z., Kemal Soylu M. (2015). Successful ram semen cryopreservation with lyophilized egg yolk-based extender. Cryobiology.

[81] Sias B., Ferrato F., Pellicer-Rubio M.T., Forgerit Y., Guillouet P., Leboeuf B., Carrière F. (2005). Cloning and Seasonal Secretion of the Pancreatic Lipase-Related Protein 2 Present in Goat Seminal Plasma. Biochim. Biophys. Acta.

[82] Iritani A., Nishikawa Y. (1963). Studies on the Egg-Yolk-Coagulating Enzyme in Goat Semen. IV. On the Position of Yolk Constituents Attacked by the Coagulating Enzyme. Jpn. J. Anim. Reprod..

[83] Roy A. (1957). Egg yolk-coagulating enzyme in the semen and Cowper’s gland of the goat. Nature.

[84] Lv C., Wu G., Hong Q., Quan G. (2019). Spermatozoa Cryopreservation: State of Art and Future in Small Ruminants. Biopreserv. Biobank..

[85] Silva S.V., Soares A.T., Batista A.M., Almeida F.C., Nunes J.F., Peixoto C.A., Guerra M.M.P. (2013). Vitamin E (Trolox) Addition to Tris-Egg Yolk Extender Preserves Ram Spermatozoon Structure and Kinematics after Cryopreservation. Anim. Reprod. Sci..

[86] Ahmet E., Ramazan A., Selin Y., İzem S.A., Nur E., Kamber D., Mithat E., Kemal A. (2025). The Effect of Cholesterol-Loaded Cyclodextrin and Resveratrol Compounds on Post-Thawing Quality of Ram Semen. Vet. Med. Sci..

[87] Suwor F., Kubota S., Nawong S., Thuangsanthia A., Toyra M., Paengkoum P., Ponchunchoovong S. (2025). Effects of Cryoprotectant Combinations on Post-Thawed Sperm Quality, Biomolecular Changes, DNA Methylation, and Pregnancy Rates in Boer Goat Semen. Vet. Sci..

[88] de Paz P., Esteso M.C., Alvarez M., Mata M., Chamorro C.A., Anel L. (2010). Development of extender based on soybean lecithin for its application in liquid ram semen. Theriogenology.

[89] Salmani H., Towhidi A., Zhandi M., Bahreini M., Sharafi M. (2014). In vitro assessment of soybean lecithin and egg yolk based diluents for cryopreservation of goat semen. Cryobiology.

[90] Schoelerman E.R., Weide T.J., Kerns K.C. (2026). Evaluation of milk-based and egg-based semen extenders for extended storage of ram semen at 4 °C. Transl. Anim. Sci..

[91] Cheng F.P., Wu J.T., Chan J.P., Wang J.S., Fung H.P., Colenbrander B., Tung K.C. (2004). The effect of different extenders on post-thaw sperm survival, acrosomal integrity and longevity in cryopreserved semen of Formosan Sika deer and Formosan Sambar deer. Theriogenology.

[92] Paulenz H., Söderquist L., Adnøy T., Fossen O.H., Berg K.A. (2003). Effect of milk- and TRIS-based extenders on the fertility of sheep inseminated vaginally once or twice with liquid semen. Theriogenology.

[93] Akhter S., Ansari M.S., Rakha B.A., Ullah N., Andrabi S.M., Khalid M. (2011). In vitro evaluation of liquid-stored buffalo semen at 5 °C diluted in soya lecithin based extender (Bioxcell^®^), tris-citric egg yolk, skim milk and egg yolk-citrate extenders. Reprod. Domest. Anim..

[94] Olivera-Muzante J., Fierro S., Gil J. (2011). Conception rates in ewes after AI with ram semen preserved in milk-egg yolk extenders supplemented with glycerol. Reprod. Domest. Anim..

[95] O’Hara L., Hanrahan J.P., Richardson L., Donovan A., Fair S., Evans A.C., Lonergan P. (2010). Effect of storage duration, storage temperature, and diluent on the viability and fertility of fresh ram sperm. Theriogenology.

[96] Liang J., Larbi A., Lv C., Ali S., Wu G., Quan G. (2023). Fertility results after exocervical insemination using goat semen cryopreserved with extenders based on egg yolk, skim milk, or soybean lecithin. Reprod. Domest. Anim..

[97] Soares A.T., Silva S.V., Batista A.M., Almeida F.C., Nunes J.F., Peixoto C.A., Guerra M.M. (2015). Ultrastructure evaluation of goat spermatozoa after freezing in a skim milk-based extender with Trolox supplementation. Andrologia.

[98] Esteve I.C., Mocé M.L., Gómez E.A., Martín-Montalbán R., Martínez-Climent A., Mocé E. (2025). The detrimental impact of seminal plasma on the quality of cryopreserved sperm in goat bucks: Freezing-thawing as the most harmful step. Front. Vet. Sci..

[99] Dorado J., Muñoz-Serrano A., Hidalgo M. (2010). The effect of cryopreservation on goat semen characteristics related to sperm freezability. Anim. Reprod. Sci..

[100] Jiménez-Rabadán P., Ramón M., García-Álvarez O., Maroto-Morales A., del Olmo E., Pérez-Guzmán M.D., Bisbal A., Fernández-Santos M.R., Garde J.J., Soler A.J. (2012). Effect of semen collection method (artificial vagina vs. electroejaculation), extender and centrifugation on post-thaw sperm quality of Blanca-Celtibérica buck ejaculates. Anim. Reprod. Sci..

[101] Kchikich A., Kirschvink N., El Kadili S., Raes M., El Otmani S., Chebli Y., Bister J.L., El Amiri B., Barrijal S., Chentouf M. (2023). Effects of *Origanum majorana* essential oil and antibiotics on the quality of frozen thawed Beni Arouss buck semen. Reprod. Domest. Anim..

[102] Kchikich A., Kirschvink N., Raes M., El Otmani S., Chebli Y., Bister J.L., El Amiri B., Barrijal S., Chentouf M. (2024). Carvacrol and Thymol Enhance the Quality of Beni Arouss Buck Semen Stored at 4 °C Thanks to Their Antimicrobial Properties. Vet. Sci..

[103] Molina J.C.J., de Oliveira M.B., Costa P.H.C., Santos E.W.P.D., Correa C.B., Azevedo H.C. (2023). Association between different soy lecithin-based extenders and freezing rates in ram semen cryopreservation. Anim. Reprod. Sci..

[104] Zhandi M., Sharafi M. (2015). Negative effect of combined cysteine and glutathione in soy lecithin-based extender on post-thawed ram spermatozoa. Cell Tissue Bank..

[105] Chelucci S., Pasciu V., Succu S., Addis D., Leoni G.G., Manca M.E., Naitana S., Berlinguer F. (2015). Soybean lecithin-based extender preserves spermatozoa membrane integrity and fertilizing potential during goat semen cryopreservation. Theriogenology.

[106] Gunawan M., Karja N.W.K., Setiadi M.A., Kaiin E.M., Said S., Arifiantini R.I., Iskandar H. (2025). Development and evaluation of soy lecithin-derived nanoliposomes as a plant-based alternative to egg-yolk extender for Ongole-grade bull semen cryopreservation. Vet. World.

[107] Krishnappa B., Binsila B.K., Arangasamy A., Gopi M., Ramachandran N., Archana S.S., Swathi D., Tomcy A.T., Ramya L., Selvaraju S. (2025). Novel insights on cryostress, cryoinjury and cryotolerance in sperm—A review. Syst. Biol. Reprod. Med..

[108] Mazenod E., Commin L., Bontemps F., Boyer L., Buff S., Pillet-Michelland E. (2026). Liposome-based extender consistently improves post-thaw semen quality in rams. Anim. Reprod. Sci..

[109] Xu C.L., Zhou J.B., Zhao B.T., Lan G.C., Luo M.J., Chang Z.L., Sui H.S., Tan J.H. (2009). Liquid storage of goat semen in chemically defined extenders. Reprod. Domest. Anim..

[110] Brito L.F.C., Linardi R.L., Rosales L.A.S., Balamurugan N.S., Hernández-Avilés C., Ramírez-Agámez L. (2025). Evaluation of a Chemically Defined, Long-Term Extender for Liquid Storage of Stallion Semen. Reprod. Domest. Anim..

[111] Salamon S., Maxwell W.M. (2000). Storage of ram semen. Anim. Reprod. Sci..

[112] Bodu M., Hitit M., Greenwood O.C., Murray R.D., Memili E. (2025). Extender development for optimal cryopreservation of buck sperm to increase reproductive efficiency of goats. Front. Vet. Sci..

[113] Bucak M.N., Ateşşahin A., Varişli O., Yüce A., Tekin N., Akçay A. (2007). The influence of trehalose, taurine, cysteamine and hyaluronan on ram semen Microscopic and oxidative stress parameters after freeze-thawing process. Theriogenology.

[114] Aires V.A., Hinsch K.D., Mueller-Schloesser F., Bogner K., Mueller-Schloesser S., Hinsch E. (2003). In vitro and in vivo comparison of egg yolk-based and soybean lecithin-based extenders for cryopreservation of bovine semen. Theriogenology.

[115] Layek S.S., Mohanty T.K., Kumaresan A., Parks J.E. (2016). Cryopreservation of bull semen: Evolution from egg yolk based to soybean based extenders. Anim. Reprod. Sci..

[116] Forouzanfar M., Sharafi M., Hosseini S.M., Ostadhosseini S., Hajian M., Hosseini L., Abedi P., Nili N., Rahmani H.R., Nasr-Esfahani M.H. (2010). In vitro comparison of egg yolk-based and soybean lecithin-based extenders for cryopreservation of ram semen. Theriogenology.

[117] Susilowati S., Sardjito T., Mustofa I., Widodo O.S., Kurnijasanti R. (2021). Effect of green tea extract in extender of Simmental bull semen on pregnancy rate of recipients. Anim. Biosci..

[118] Ros-Santaella J.L., Pintus E. (2021). Plant Extracts as Alternative Additives for Sperm Preservation. Antioxidants.

[119] Watson P.F. (2000). The causes of reduced fertility with cryopreserved semen. Anim. Reprod. Sci..

[120] Mocé E., Purdy P.H., Graham J.K. (2010). Treating ram sperm with cholesterol-loaded cyclodextrins improves cryosurvival. Anim. Reprod. Sci..

[121] Abbasi R., Farhadi R., Varjovi S.G., Najafi A. (2026). Effect of nanoliposomal gamma-oryzanol on cryosurvival, oxidative balance, and fertility of frozen-thawed ram spermatozoa. Sci. Rep..

[122] Kumar P., Saini M., Kumar D., Balhara A.K., Yadav S.P., Singh P., Yadav P.S. (2015). Liposome-based semen extender is suitable alternative to egg yolk-based extender for cryopreservation of buffalo (*Bubalus bubalis*) semen. Anim. Reprod. Sci..

[123] Luna-Orozco J.R., González-Ramos M.A., Calderón-Leyva G., Gaytán-Alemán L.R., Arellano-Rodríguez F., Ángel-García O., Véliz-Deras F.G. (2019). Comparison of different diluents based on liposomes and egg yolk for ram semen cooling and cryopreservation. Iran. J. Vet. Res..

[124] Saadeldin I.M., Khalil W.A., Alharbi M.G., Lee S.H. (2020). The Current Trends in Using Nanoparticles, Liposomes, and Exosomes for Semen Cryopreservation. Animals.

[125] Kaya C., Can C., Esin B., Dünder E., Çevik M., Akar M. (2026). Effects of Selenium Nanoparticles and Sodium Selenite Supplementation on Cryopreserved Ram Sperm Quality, Oxidative Status, and PRDX5 Gene Expression. Animals.

[126] Salimi T., Hajarian H., Karamishabankareh H., Soltani L. (2024). Effects of sodium selenite, cysteamine, bacterially synthesized Se-NPs, and cysteamine loaded on Se-NPs on ram sperm cryopreservation. Sci. Rep..

[127] Öztürk A.E., Bodu M., Atay Y.E., Sarıözkan S., Şahin D., Korkmaz O., Öçsoy İ., Hitit M. (2026). The Effects of Graphene Oxide Nanoparticles on the Cryopreservation of Angora Buck Sperm. Molecules.

[128] Öztürk A.E., Sarıözkan S., Bodu M., Atay Y.E., Şahin D., Korkmaz O., Öztürk C., Kılınç Z., Mirioglu M., Temur N. (2026). Size-dependent cryoprotective effects of curcumin nanoparticles on DNA integrity and motility in cryopreserved Angora buck sperm. Front. Vet. Sci..

[129] Deller R.C., Vatish M., Mitchell D.A., Gibson M.I. (2014). Synthetic polymers enable non-vitreous cellular cryopreservation by reducing ice crystal growth during thawing. Nat. Commun..

[130] Faigl V., Vass N., Jávor A., Kulcsár M., Solti L., Amiridis G., Cseh S. (2012). Artificial insemination of small ruminants—A review. Acta Vet. Hung..

[131] Ritar A.J., Salamon S. (1982). Effects of Seminal Plasma and of Its Removal and of Egg Yolk in the Diluent on the Survival of Fresh and Frozen-Thawed Spermatozoa of the Angora Goat. Aust. J. Biol. Sci..

[132] Leboeuf B., Restall B., Salamon S. (2000). Production and storage of goat semen for artificial insemination. Anim. Reprod. Sci..

[133] Mata-Campuzano M., Álvarez-Rodríguez M., Álvarez M., Tamayo-Canul J., Anel L., de Paz P., Martínez-Pastor F. (2015). Post-thawing quality and incubation resilience of cryopreserved ram spermatozoa are affected by antioxidant supplementation and choice of extender. Theriogenology.

[134] Akourki A., Mondal M., Karunakaran M., Pal P. (2018). Effect of soybean lecithin extender on post-thaw semen quality of Bengal buck. Indian J. Anim. Health.

[135] Gourley D.D., Riese R.L. (1990). Laparoscopic artificial insemination in sheep. Vet. Clin. N. Am. Food Anim. Pract..

[136] Hiwasa M., Kohno H., Togari T., Okabe K., Fukui Y. (2009). Fertility after different artificial insemination methods using a synthetic semen extender in sheep. J. Reprod. Dev..

[137] Bisla A., Honparkhe M., Srivastava N. (2022). A review on applications and toxicities of metallic nanoparticles in mammalian semen biology. Andrologia.

[138] Gad W.A., Ibrahim S., Nagdy H., Elsawy B.S.M., Aboelsoued D., Abdel-Ghany H.S.M., Abdel-Wareth A.A.A., Abd El-Razik K.A., Mahmoud K.G.M., Soliman W.T.M. (2025). Bridging biology and therapy: Translational advances of extracellular vesicles in veterinary clinical practice. Vet. Res. Commun..

[139] Capra E., Frigerio R., Lazzari B., Turri F., Gaspari G., Pascucci L., Stella A., Lange Consiglio A., Pizzi F., Cretich M. (2024). Effect of cryopreservation and semen extender on extracellular vesicles isolated from bull semen. Front. Vet. Sci..

[140] Urli S., Corte Pause F., Dreossi T., Crociati M., Stradaioli G. (2025). Evaluation of an artificial intelligence system for bull sperm morphology evaluation. Theriogenology.

